# Sublingual immune cell clusters and dendritic cell distribution in the oral cavity

**DOI:** 10.1172/jci.insight.167373

**Published:** 2024-10-01

**Authors:** Yutaka Kusumoto, Mizuki Ueda, Mayuko Hashimoto, Haruka Takeuchi, Naoko Okada, Junya Yamamoto, Akiko Nishii, Atsuki Fujino, Akiho Kurahashi, Momoka Satoh, Yuki Iwasa, Koki Okamura, Karin Obazaki, Ryoto Kumagai, Naruya Sakamoto, Yuto Tanaka, Yukika Kamiya, Tetsushi Hoshida, Tsuneyasu Kaisho, Hiroaki Hemmi, Tomoya Katakai, Tetsuya Honda, Junichi Kikuta, Kosuke Kataoka, Ryoyo Ikebuchi, Taiki Moriya, Takahiro Adachi, Takeshi Watanabe, Masaru Ishii, Atsushi Miyawaki, Kenji Kabashima, Tatyana Chtanova, Michio Tomura

**Affiliations:** 1 Laboratory of Immunology, Faculty of Pharmacy, Osaka Ohtani University, Tondabayashi, Osaka, Japan.; 2 Laboratory for Cell Function Dynamics, RIKEN Center for Brain Science, Wako, Saitama, Japan.; 3 Biotechnological Optics Research Team, RIKEN Center for Advanced Photonics, Wako, Saitama, Japan.; 4 Department of Immunology, Institute of Advanced Medicine, Wakayama Medical University, Graduate School of Medicine, Wakayama, Wakayama, Japan.; 5 Laboratory of Immunology, Faculty of Veterinary Medicine, Okayama, University of Science, Imabari, Ehime, Japan.; 6 Department of Immunology, Graduate School of Medical and Dental Sciences, Niigata University, Niigata, Niigata, Japan.; 7 Department of Dermatology, Kyoto University, Graduate School of Medicine, Sakyo-ku, Kyoto, Japan.; 8 Department of Dermatology, Hamamatsu University School of Medicine, Handayama, Hamamatsu, Japan.; 9 Laboratory of Immunology and Cell Biology, Graduate school of Medicine, Osaka University, Suita, Osaka, Japan.; 10 Department of Oral Health Science and Social Welfare, Graduate school of Oral Sciences, Tokushima University, Tokushima, Tokushima, Japan.; 11 Research Fellow of Japan Society for the Promotion of Science, Japan.; 12 Department of Precision Health, Medical Research Institute, Tokyo Medical and Dental University, Tokyo, Japan.; 13 Laboratory of Immunology, Institute for Life and Medical Sciences, Kyoto University, Sakyo-ku, Kyoto, Japan.; 14 School of Biotechnology and Biomolecular Sciences, Faculty of Science, University of New South Wales Sydney, Kensington, New South Wales, Australia.; 15 Immunology Theme, Garvan Institute of Medical Research, Darlinghurst, New South Wales, Australia.

**Keywords:** Immunology, Dendritic cells, T cells

## Abstract

The oral mucosa is the first line of defense against pathogenic bacteria and plays a vital role in maintaining tolerance to food antigens and commensal bacteria. We used CD11c reporter mice to visualize dendritic cells (DCs), a key immune cell population, in the oral cavity. We identified differences in DC density in each oral tissue region. Sublingual immune cell clusters (SLICs) extended from the lamina propria to the epithelium, where DCs and T cells resided in close contact with each other and innate lymphoid cells. Targeted in situ photolabeling revealed that the SLICs comprised mostly CD11c^+^CD11b^+^ DCs and were enriched for cDC1s and Langerhans cells. Although the frequency of T cell subsets was similar within and outside the SLICs, tissue-resident memory T cells were significantly enriched within the clusters and cluster size increased in response to inflammation. Collectively, we found that SLICs form a unique microenvironment that facilitates T cell–DC interactions in the steady state and during inflammation. Since the oral mucosa is an important target for needle-free vaccination and sublingual immunotherapy to induce tolerogenic responses, the insight into the localized immunoregulation provided in this study may accelerate the development of these approaches.

## Abstract 2

Sublingual immune cell clusters were enriched for cDC1s and Langerhans cells and TRMs and cluster number and size were increased in response to inflammation.

## Graphical abstract

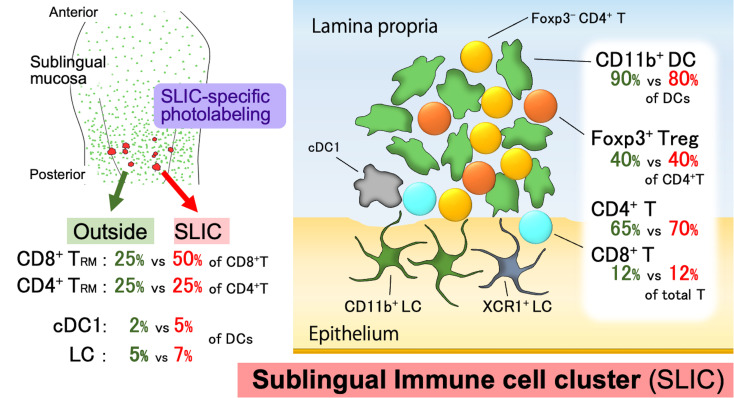

## Introduction

The oral cavity is the first part of the digestive system that comes into contact with foreign antigens. Immune cells in the oral cavity form the first line of defense against pathogenic bacteria and maintain oral tolerance to food antigens and commensal bacteria (1, 2). They present potential targets for therapeutic regulation of systemic immune responses by painless needle-free vaccination and sublingual immunotherapy (SLIT) for type I allergic diseases (3–6). Dendritic cells (DCs) in peripheral tissues serve as sentinels for exogenous antigens, which they capture and present to T cells in the draining lymph nodes (dLNs) to initiate adaptive immune responses (7). DCs can also contact and directly stimulate T cells in peripheral tissues (8). Thus, information regarding the distribution and function of DCs in the oral mucosa is crucial for understanding the role of the oral immune system and how it mediates systemic immunity (7).

The oral surface layer comprises, in order from the surface, a parakeratinized or non-keratinized epithelium, basal layer, and lamina propria (LP) (6). Langerin^+^ (CD207)^+^ Langerhans cells (LCs) reside within the epithelium (9). Recently, it was reported that unlike the skin epidermis, where only the CD11b^+^CD103^–^ subset is present, LCs in the buccal and gingival mucosa contain both CD103^+^CD11b^lo^ and CD11b^+^CD103^–^ subsets differentiated from pre-DCs and monocytic precursors (10). While LCs also exist in sublingual epithelium, their phenotype is unclear.

In the buccal LP, in addition to interstitial CD11b^+^CD103^–^ DCs, langerin^+^CD103^+^ and langerin^–^CD103^+^ DCs are also present (11). These CD103^+^DCs (called type 1 conventional DCs: cDC1s) directly present exogenous antigens to CD8^+^ T cells (12–14). In the sublingual mucosa, CD11b^+^ CD103^–^ DCs are the major DC subset capable of inducing Foxp3^+^ T cells after migration to dLNs (15). Notably, vaccination in the sublingual region induces systemic CD8^+^ T cell responses (16, 17). Thus, it is important to identify the phenotype and distribution of the sublingual DCs to clarify the role of the sublingual region in oral and systemic immunity.

Certain DCs in the oral mucosa enhance immune responses, while others contribute to immune tolerance (9, 15, 18–21). Despite several studies focusing on oral DC subsets, these studies mainly employed immunohistochemistry, which accesses only small sections of tissues, and flow cytometry, which does not provide 3-dimensional (3D) information about tissues. Therefore, insufficient information is available about DC distribution and localization in the oral mucosa.

The mucosa is subdivided into type I and type II based on its structure (8, 22). Type I mucosa is the monolayered epithelium. It covers the surface of the intestinal tract, nasal mucosa, and bronchus. Mucosal-associated lymphoid tissues (MALTs) can be found in these structures. MALTs contain DCs and naive T and B cells, and serve as sites of antigen presentation to naive T cells and sensitization of B cells in peripheral tissues (2). On the other hand, type II mucosa is the stratified epithelium and covers the surface of the oral cavity, genital mucosa, and skin (8, 22). Until recently, MALT-like immunological foci have not been detected in type II mucosa (8, 22). However, recent studies identified clusters containing DCs, macrophages, and T cells around hair follicles in intact skin of the flank (8, 23). In addition, inducible skin-associated lymphoid tissue (iSALT), which includes DCs and memory T cells, particularly, tissue-resident T cells (Trms), was identified in ear skin in an experimental model mouse of contact hypersensitivity (24, 25). Similar inducible clusters are present in the vaginal mucosa and lung parenchyma (26–28). These clusters are thought to play important roles in defense against exogenous pathogens and mediate secondary adaptive immune responses in peripheral tissues in the steady state and during immune responses (8, 25, 29). Therefore, determining whether such clusters exist in the oral cavity is important for elucidating the role of the local mucosa in oral immunity.

Here we used a transgenic mouse line in which CD11c promoter–driven yellow fluorescent protein (CD11c-YFP) is expressed in DCs (30) in combination with tissue-clearing technology to visualize their distribution throughout the oral mucosa. We show that fluorescent DCs were found in compartment-specific distribution patterns or clusters. These clusters in the sublingual region also contained T cells (and were named sublingual immune cell clusters, or SLICs). We observed that DCs and T cells were in close contact with each other. By using cluster-specific photolabeling with the photoconvertible protein KikGR, we discovered that SLICs comprised mostly CD11c^+^CD11b^+^ DCs and had a higher proportion of cDC1s and LCs than outside the clusters. T cells within the SLICs were composed largely of regulatory CD4^+^ T cells (Tregs) and some CD8^+^ T cells of the memory/effector phenotype. The proportion of Trms was enriched in the SLICs and cluster number and size were increased in response to inflammation.

## Results

### Distinct DC densities within regions of the oral cavity.

CD11c-YFP reporter mice allow detection of DCs as YFP^+^ cells (30). We first determined the specificity of YFP expression in DCs in the oral mucosa. Flow cytometric analysis of cells from the sublingual region of CD11c-YFP mice showed that greater than 94% of YFP^+^ cells also expressed DC markers CD11c and MHC class II. When CD11c^+^MHC class II^+^ sublingual cells were gated, 80% of these cells expressed YFP, indicating that most, but not all, DCs were YFP^+^ (Supplemental Figure 1; supplemental material available online with this article; https://doi.org/10.1172/jci.insight.167373DS1). YFP^+^ cells were observed at a depth of greater than 70 μm in all intact oral tissues, except for the dorsal tongue, although the observable depth depended on the specific region (Supplemental Figure 2). We noted that although autofluorescence appeared higher in CD11c-YFP mice than in WT mice, YFP^+^ cells could be detected regardless of autofluorescence in CD11c-YFP mice (Supplemental Figure 3).

**Figure 1:**
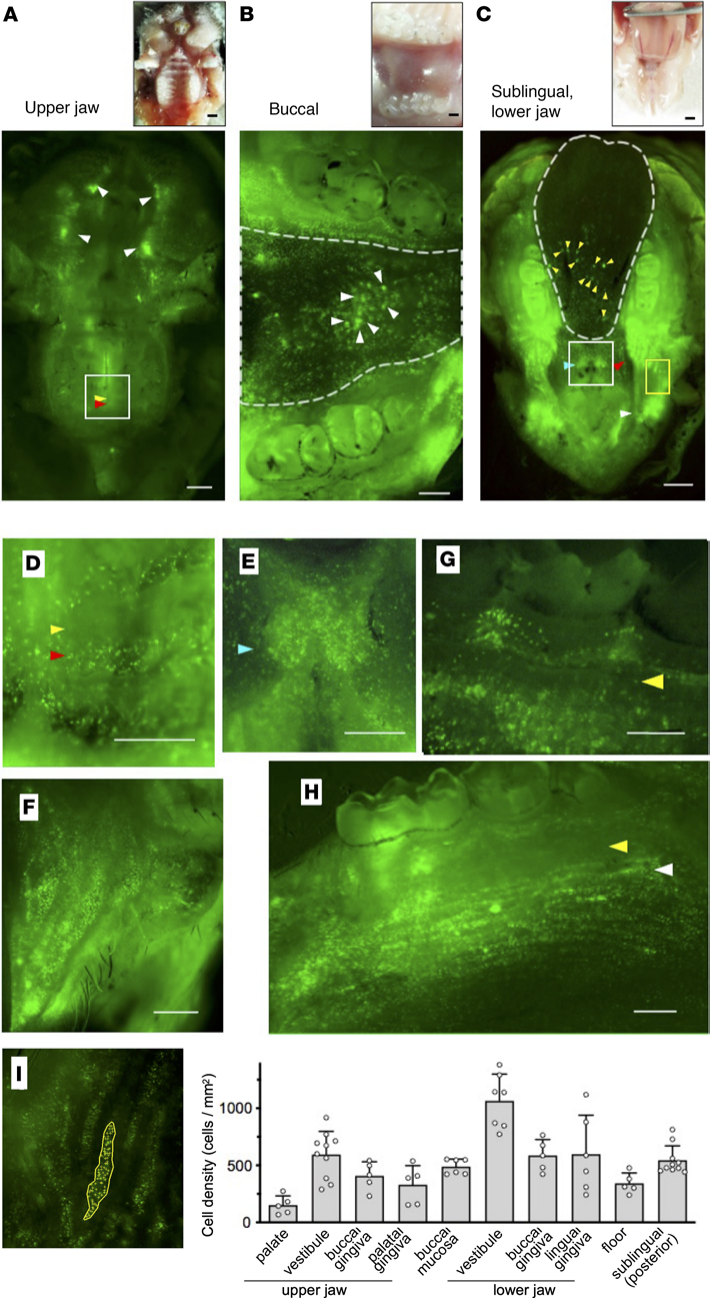
Distinct distribution patterns of DCs in the oral cavity of CD11c-YFP mice. (**A**–**C**) Bright-field images (upper panels) and fluorescence images (lower panels) acquired using fluorescence stereoscopic microscopy. (**A**) Upper jaw. White, yellow, and red arrowheads in lower panel point to the oral vestibules, palate folds, and the intermediate region, respectively. (**B**) Buccal mucosa. The region enclosed by the dotted line indicates the buccal mucosa and arrowheads point to high-density DC spots (lower panel). (**C**) Sublingual mucosa and oral floor. The region enclosed by the dotted line indicates the sublingual mucosal surface and white, red, and light blue arrowheads point to the vestibules, the alveolar ridge in front of the molars, and the caruncle, respectively (lower panel). Yellow arrowheads point to high-density DC spots in the sublingual region. (**D**–**H**) Fluorescence images acquired using fluorescence stereoscopic microscopy. (**D**) Magnified image of the area enclosed in the white box in **A**. Yellow and red arrowheads point to the palate folds and the intermediate region, respectively. (**E**) Magnified image of the area enclosed in the white box in **C**. Light blue arrowhead points to the caruncle. (**F**) Magnified image of the area enclosed in the yellow box in **C**. (**G**) Buccal gingiva. Yellow arrowhead points to the attached gingiva. (**H**) Lingual gingiva. Yellow and white arrowheads point to the attached gingiva and mucogingival junction, respectively. (**I**) The number of DCs in each region of the oral cavity in which the DCs accumulated (left) was assessed and calculated as the DC density of each region of the oral cavity (right). Data represent mean ± SEM (*n* = 5). The data are representative of at least 3 independent experiments. CD11c-YFP is stained green. Scale bars: 1000 μm (**A** and **C**) and 500 μm (**B** and **D**–**H**).

Next, we visualized the distribution of YFP^+^ DCs in intact tissues of the upper jaw, buccal, sublingual, and lower jaw (Figure 1). DC density was relatively low in the palate and palatal gingiva of the upper jaw (Figure 1, A and I). In contrast, high DC density regions (marked by arrowheads) were observed in the vestibule, buccal, sublingual, and lower jaw (Figure 1, A, B, C, and I). On the other hand, we did not observe YFP^+^ cells on the surface of the dorsal tongue (Supplemental Figure 4).

### Distinct DC distribution patterns in the oral mucosa.

We next examined the distribution of DCs in each region of the oral cavity. More YFP^+^ cells were present in the oral vestibules (Figure 1A, marked by white arrowheads), maxilla (Figure 1A), and mandible (Figure 1C). In the palate of the maxilla, YFP^+^ cells were present between palate folds (red arrowheads in Figure 1, A and D). In addition, YFP^+^ cells were observed in the maxillary buccal gingiva, but not in the palatal gingiva, and were infrequent in the alveolar ridge (Figure 1A).

In the mandible, numerous YFP^+^ cells were present in the caruncle near the saliva outlets from the submandibular and sublingual glands (light blue arrowheads in Figure 1, C and E). A high density of YFP^+^ cells was observed in the alveolar ridge of the region from the molars to the vestibule (red and white arrowheads in Figure 1C), but not in the zonal region of the attached gingiva (yellow arrowheads, Figure 1, G and H). Highly dense single YFP^+^ cells were distributed in stripes, particularly in the mandible lingual gingival mucosa (white arrowhead, Figure 1H). Furthermore, isolated single YFP^+^ cells and high-density YFP^+^ cell spots were observed in the buccal mucosa (arrowheads in dotted line region in Figure 1B) and in the sublingual surface (yellow arrowheads in posterior half of dotted line region in Figure 1C).

We counted the number of DCs to quantify DC density in each region (Figure 1I). DC densities in lower jaw trended higher compared with those in the upper jaw. DC densities (mean cells/mm^2^ in each area) were the highest in the vestibular area (1063.8 and 594.0 cells/mm^2^ in the lower and upper jaw, respectively), followed by the gingiva (buccal, 586.1 cells/mm^2^ and lingual, 598.0 cells/mm^2^) and the floor (342.5 cells/mm^2^) in the lower jaw, whereas they were lower in the gingiva (buccal, 408.1 cells/mm^2^ and palatal, 329.6 cells/mm^2^) and lowest in the palate (151.0 cells/mm^2^) in the upper jaw. DC densities in the buccal mucosa (489.0 cells/mm^2^) and sublingual (544.6 cells/mm^2^) mucosa were moderate.

In summary, YFP^+^ cells were distributed throughout the mandibular, but not the maxillary, mucosa, and their distribution patterns were distinct within each region (Table 1). High densities of single DCs were observed in the mucosal tissues of the vestibule as well as in the alveolar ridge in front of the lower molars. Further, high-density DC spots were observed in the buccal and sublingual mucosae.

### DCs are distributed in the dorsal lingual mucosa with filiform papillae.

**Figure 2:**
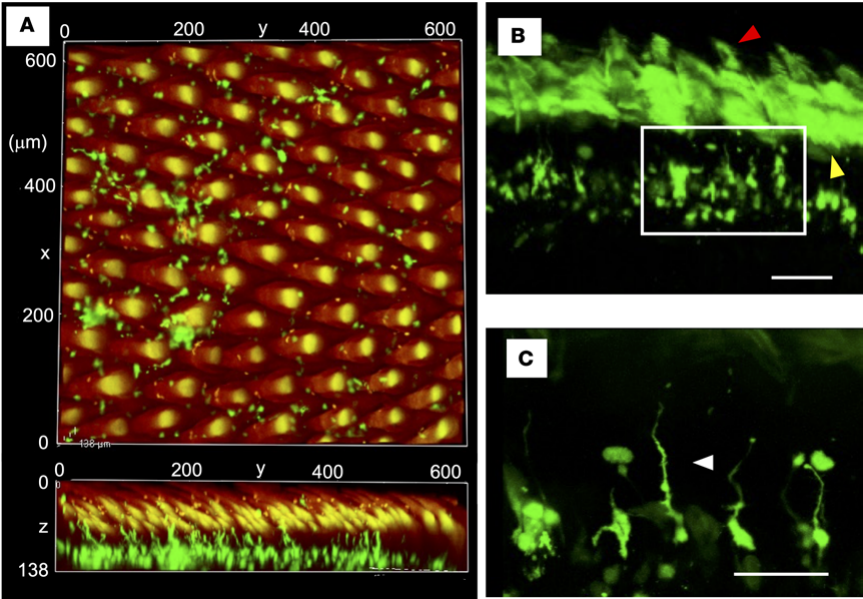
DCs in the dorsal tongue. Images of the dorsal tongue acquired using confocal laser microscopy. (**A**) The posterior dorsal surface of the tissue-cleared tongue was observed. Upper: vertical image. Lower: sagittal image (Supplemental Video 1). (**B**) The surface of the coronal section of the tissue-cleared tongue was observed. Yellow and red arrowheads in **B** point to the autofluorescence of epithelium and filiform papillae, respectively. (**C**) Enlarged image of the area enclosed by the white square in **B**. Green, CD11c-YFP. Red, autofluorescence. The arrowhead points to the elongated dendrite to the cryptic bottoms on the dorsal surface. Scale bars: 100 μm (**B**) and 50 μm (**C**).

Since YFP^+^ cells on the dorsal surface of the intact tongue were undetectable using fluorescence microscopy (Supplemental Figure 4), we performed the tissue-clearing technique Sca*l*eS (31) to examine the tongue. Using this approach, we could observe up to 150 μm below the surface of the tongue and detected DCs scattered in the dorsal lingual mucosa with filiform papillae (Figure 2, A and B, and Supplemental Video 1). Their dendrites extended to the cryptic bottoms among the papillae (Figure 2 and Supplemental Video 1, white arrowhead in Figure 2C). Fewer YFP^+^ cells were found in the anterior region than in the posterior region (Figure 2A, Supplemental Figure 5, and Supplemental Video 1).

### Single DCs are scattered in the basal layer of rete pegs in the buccal mucosa.

**Figure 3:**
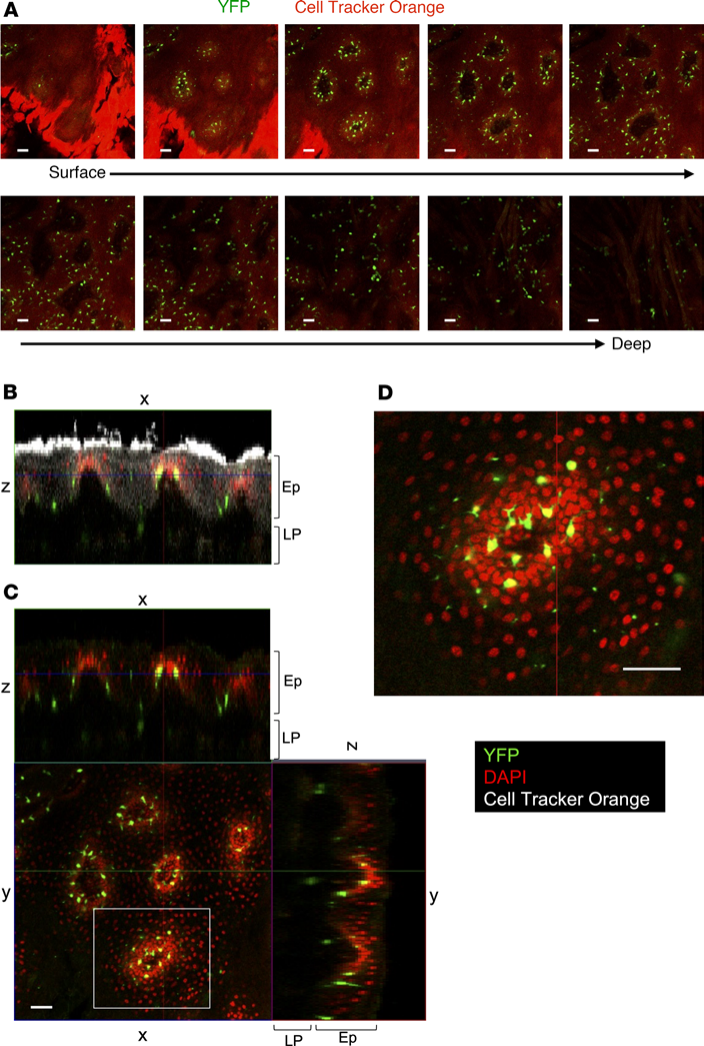
Single DCs scattered in the basal layer of rete pegs in the buccal mucosa. The CD11c^+^ cells in the buccal mucosa were observed using confocal microscopy. (**A**) *Z*-stack images from the mucosal surface to muscle layer. Green, CD11c-YFP. Red, Cell Tracker Orange. (**B** and **C**) Orthogonal view of the buccal mucosa: *x*–*z* image (**B**) and *x*–*z*, *x*–*y*, and *y*–*z* images (**C**). (**D**) High magnification of the image enclosed by the white square in **C**. Green, YFP. Red, DAPI. White, Cell Tracker Orange. The data are representative of at least 3 independent experiments. Scale bars: 50 μm. LP, lamina propria; Ep, epithelium.

We observed high-density DC spots within the central region of the buccal mucosa (arrowhead in Figure 1B). Using confocal microscopy on cleared tissue, we examined buccal mucosal tissues in 3D. Analysis of *Z*-stack sections from the mucosal surface down to muscle revealed YFP^+^ cells forming rings in the basal layer of the rete peg (Figure 3). Thus, single DCs scattered through the basal layer of rete pegs appeared to form high-density DC spots when observed from outside of the tissue.

### Single DCs and DC clusters in the sublingual mucosa.

**Figure 4:**
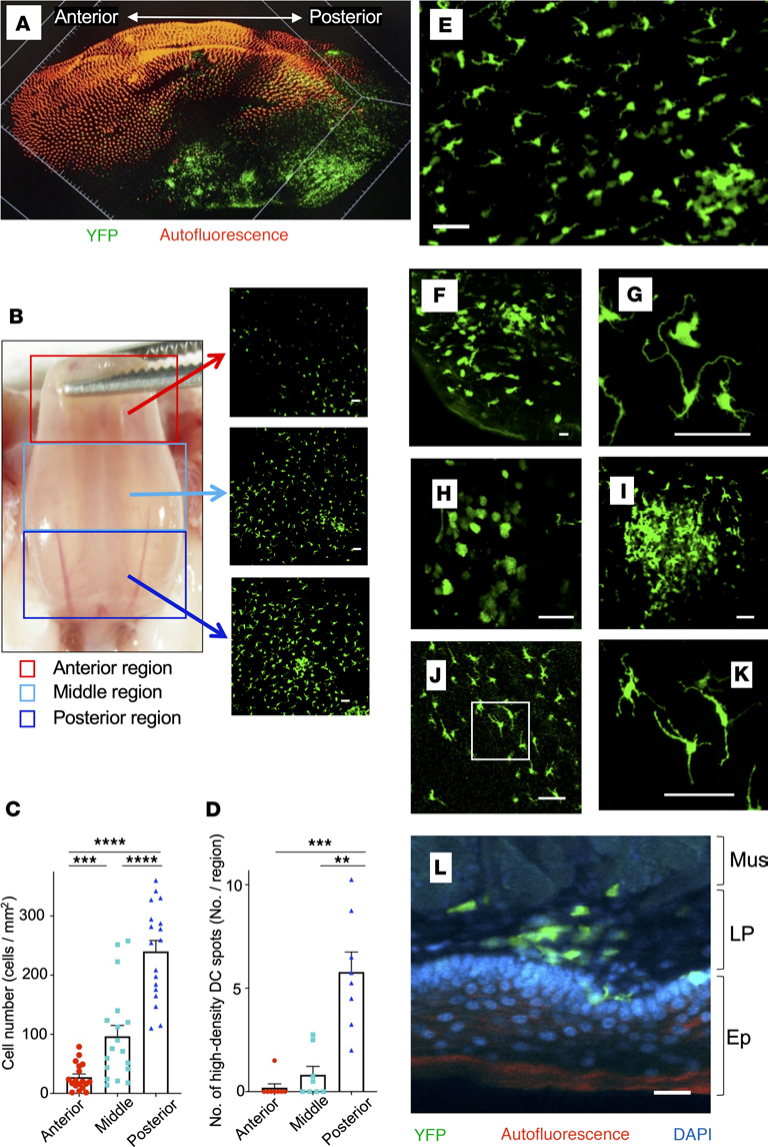
Single DCs and DC clusters in the posterior region of the sublingual mucosa. (**A**) Image of the entire tissue-cleared tongue of CD11c-YFP mouse acquired using a light-sheet microscope (orange signal is autofluorescence of surface of tongue). (**B**) Bright-field (left panel) and fluorescence (right panel) images of the sublingual mucosa of CD11c-YFP mouse. Fluorescence images of the anterior (red square), middle (light-blue square), and posterior (blue square) regions of the sublingual surface. (**C** and **D**) Numbers of single DCs and DC clusters in the 3 subdivided regions in **B**. Data represent mean ± SD, single DCs (*n* = 18), DC clusters (*n* = 8). Statistical comparisons were performed using 1-way ANOVA with Tukey’s multiple-comparison test. **P < 0.01; ***P < 0.001; ****P < 0.0001. (**E**–**I**) Images of tissue-cleared sublingual mucosa of CD11c-YFP mice were acquired using confocal microscopy. Sublingual mucosa (**E** and **F**), DCs in the epithelium (**G**) (Supplemental Video 2), single DCs (**H**), and a DC cluster (**I**) (Supplemental Video 3) in the LP. (**J** and **K**) Fluorescence images of DCs in the sublingual posterior region in Langerin-Cre/KikGR mice. The white square in **J** was further magnified and shown in **K**. (**L**) Fluorescence image of the coronally cut surface of the sublingual tongue. Green, CD11c-YFP. Red, autofluorescence. Blue, DAPI. The data are representative of at least 3 independent experiments. Scale bars: 50 μm (**B** and **E**–**K**) and 25 μm (**L**). Mus, muscle layer; LP, lamina propria; Ep, epithelium.

We noted high-density DC spots in the sublingual mucosa (region enclosed by the dotted line in Figure 1C). To obtain an overall picture of DC distribution in the sublingual region, we first examined tissue-cleared whole tongue using light-sheet microscopy. Using this approach, we detected significantly more YFP^+^ cells in the posterior part of the sublingual region (Figure 4A). Accordingly, we subdivided the sublingual area into 3 regions — anterior, middle, and posterior (the regions beside the junction between the sublingual surface and oral floor) — and quantitated scattered single YFP^+^ cells and high-density DC spots in each region (Figure 4B). Single YFP^+^ cell density in the sublingual mucosa significantly increased toward the posterior region, with a 6-fold greater DC density in the posterior region compared with the anterior region (Figure 4C). Additionally, high-density DC spots were present mostly in the posterior region (Figure 4D). We also examined changes in cluster formation over time (Supplemental Figure 6). Sublingual clusters were not observed in most 3-week-old CD11c-YFP mice, but could be observed in some 6-week-old mice, and were present in most 10-week-old mice. The number of clusters stabilized by the age of 15 weeks. No significant differences were observed between males and females.

Observations of cleared sublingual epithelium revealed YFP^+^ cells with long dendrites (Figure 4, E–G, and Supplemental Video 2). In langerin reporter mice (where the photoconvertible protein KikGR is expressed in langerin^+^ cells such as LCs), langerin^+^ cells with elongated dendrites were scattered in the sublingual mucosa (Figure 4, J and K). Based on this, we concluded that YFP^+^ cells with long dendrites that we observed in the epidermis were LCs, consistent with the previous reports (9). In addition, single YFP^+^ cells with short and few or no dendrites were observed in the LP (Figure 4, E and H, and Supplemental Figure 2). These shapes are similar to dermal DCs in the skin. Furthermore, high-density DC spots comprised densely packed DCs with short dendrites (Figure 4, E, F, and I, and Supplemental Video 3). These high-density DC spots, which we called “DC clusters,” were mainly located in the LP of the mucosa, with some spreading to the epithelial layer (Figure 4L).

### DC subsets in sublingual DC clusters.

**Figure 5:**
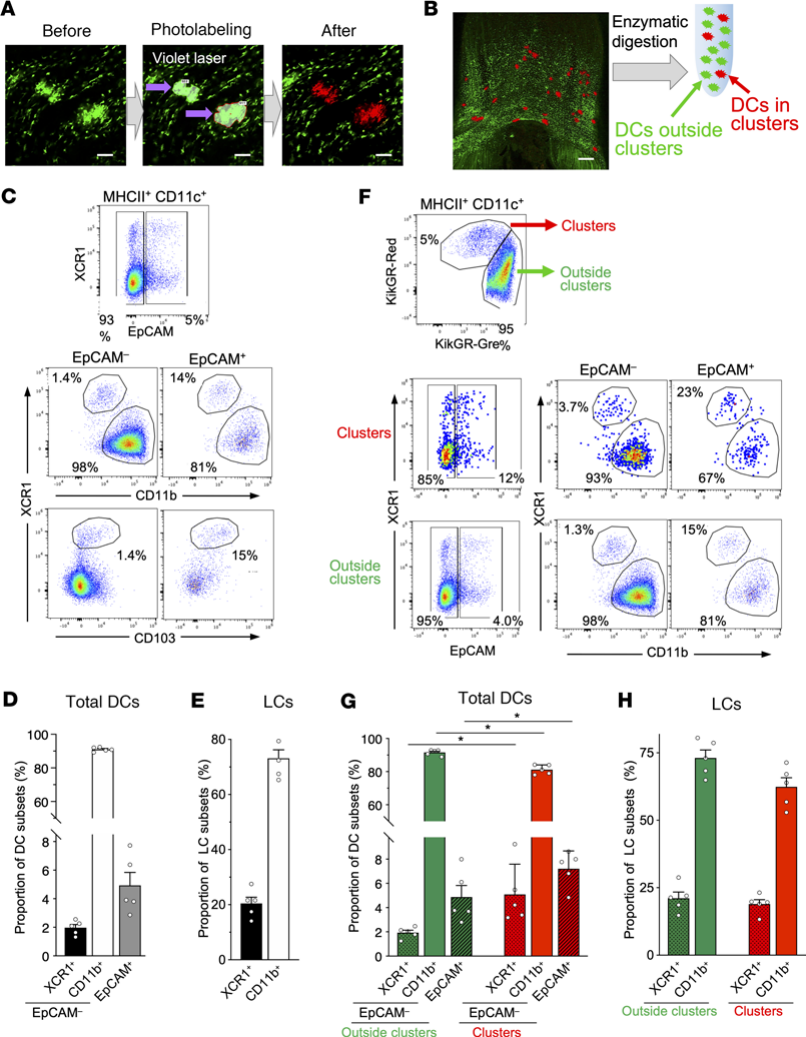
DC subsets outside and within sublingual clusters. (**A**) Photolabeling of sublingual DC clusters in CD11c-KikGR mouse. KikGR^+^ clusters in the sublingual region were surrounded with an ROI and irradiated with violet light. (**B**) Single cells were prepared from sublingual DC clusters labeled with KikGR-Red. (**C**–**H**) Single-cell suspensions prepared in **B** were stained with fluorochrome-conjugated antibodies. (**C**) Representative flow cytometry plots of KikGR^+^CD11c^+^ DCs in sublingual mucosa of CD11c-KikGR mice. (**D** and **E**) Frequencies of DC subsets in total DCs and in LCs in sublingual mucosa of CD11c-KikGR mice. (**F**) Representative flow cytometry plots of KikGR^+^CD11c^+^ DCs and photolabeled KikGR-Red DCs in clusters and non-photolabeled DCs outside clusters in sublingual mucosa of CD11c-KikGR mice. (**G** and **H**) Frequencies of DC subsets in total DCs and in LCs inside or outside clusters of sublingual mucosa of CD11c-KikGR mice. Data in **D**, **E**, **G**, and **H** represent mean ± SEM (*n* = 5). Statistical comparisons were performed using 1-way ANOVA with Tukey’s multiple-comparison test. *P < 0.05. The data are representative of at least 3 independent experiments. Scale bars: 50 μm (**A**) and 500 μm (**B**).

Since several DC subsets populate the oral cavity (9, 10, 15, 18–21), we next sought to identify which DC subsets contribute to the sublingual DC clusters. Because these clusters are microscopic in size, it is technically challenging to excise them in order to identify their constituents. To overcome this limitation, we took advantage of the photoconversion system to label only the cluster-infiltrating cells using microscopically targeted in situ photoconversion, thereby distinguishing them from the cells outside the clusters (32). Specifically, to differentiate DCs within a cluster from DCs outside of the clusters, we generated CD11c-KikGR mice in which DCs express the photoconvertible protein KikGR, and then performed cluster-specific photolabeling (Figure 5A and Supplemental Figure 7A). Using confocal microscopy, we identified KikGR^+^ clusters in the sublingual mucosa, marked the clusters as regions of interest (ROIs) and irradiated the ROIs with violet laser light to specifically convert the DCs inside the clusters to KikGR-Red (Figure 5A). Following photoconversion, all cells in the sublingual mucosa were isolated by enzymatic treatment and analyzed by flow cytometry (Figure 5B). DC subsets can be distinguished using specific cell surface markers; e.g., LCs coexpress langerin and EpCAM (33). Capucha et al. identified EpCAM^+^ cells as LCs in the gingiva and buccal mucosae and reported that LCs consist of CD11b^+^ and CD103^+^ phenotypes (10). In addition, XCR1 expression has been found to be more specific to cDC1s than CD103 (7, 13, 14, 34), especially in the mucosal layer (35). Thus, we first gated EpCAM^+^ cells as LCs and then identified CD11b^+^ DCs and XCR1^+^CD11b^–^ cDC1s in the EpCAM^–^ population. Sublingual DCs consisted of approximately 2% cDC1s, 90% CD11b^+^ DCs, and approximately 5% LCs (Figure 5, C–E, and Supplemental Figure 7B). Thus, CD11b^+^ DCs were the most abundant subset of sublingual DCs, in line with previous studies (9). Almost 20% of LCs expressed XCR1^+^, while the rest were CD11b^+^ (Figure 5E). Like XCR1^+^ cDC1s, XCR1^+^ LCs also coexpressed CD103 (Figure 5C). Specific phenotypes and biological roles of DC subsets in humans and mice are summarized in Supplemental Table 1) (7, 36–40).

Next, we compared the composition of photolabeled KikGR-Red DC clusters and non-photolabeled KikGR-Green DCs outside of the clusters. DCs in the KikGR-Red^+^ clusters accounted for approximately 3%–5% of total KikGR^+^ DCs (Figure 5F). Both within and outside of the clusters, CD11b^+^ DCs were the main DC subset (Figure 5, F and G). However, DC clusters were enriched for cDC1s and LCs compared with the DC populations outside of the clusters (Figure 5, F and G). This indicates that the clusters form unique immune foci enriched for specific DC subsets. Within the LC population, approximately one-fifth of LCs were XCR1^+^ cells and the rest were CD11b^+^, both within and outside of the clusters (Figure 5H).

### XCR1^+^ DC distribution in the sublingual mucosa.

**Figure 6:**
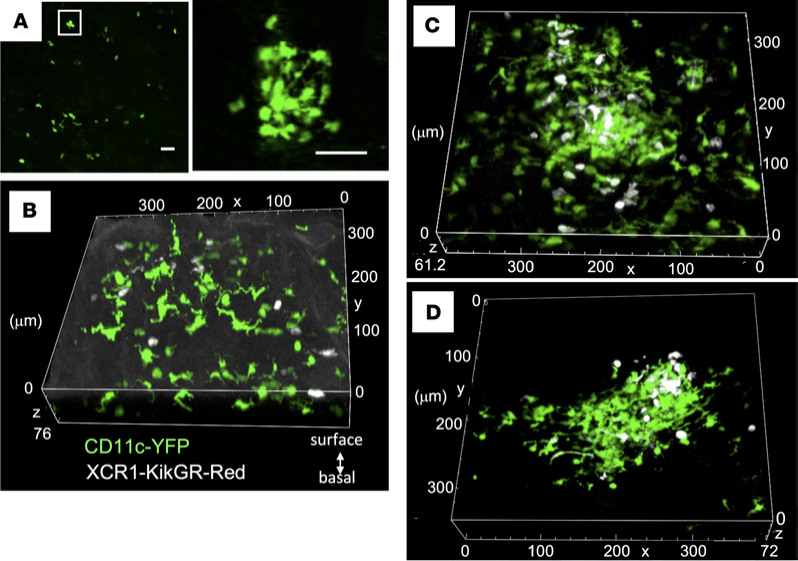
Distribution of XCR1^+^ DCs in the sublingual posterior region. (**A**) Fluorescence images of XCR1^+^ DCs in the sublingual posterior region in XCR1-KikGR mice. The white squares in the left panels were further magnified and shown in the right panel. (**B**–**D**) Confocal images of the sublingual mucosa of CD11c-YFP/XCR1-KikGR mice after exposure to violet light to photoconvert KikGR-expressing cells to KikGR-Red. KikGR-Red cells are shown in white (Supplemental Videos 4–6). The data are representative of at least 3 independent experiments. Scale bars: 50 μm (**A**).

Since cDC1s play a key role in presenting antigens to CD8^+^ T cells, we decided to investigate their distribution in the sublingual region, and especially the location of cDC1s within clusters. We examined XCR1-KikGR mice in which XCR1^+^ DCs (including cDC1s in peripheral tissues) express KikGR (41) and observed the presence of KikGR^+^ cells with few or no dendrites in the sublingual mucosa. Although there were fewer clusters in XCR1-KikGR^+^ mice compared with CD11c-YFP mice, we reproducibly detected clusters formed by rounded KikGR^+^ cells in the posterior region of the tongue (Figure 6A).

To examine the distribution of XCR1^+^ DCs, including cDC1s, in more detail, we generated CD11c-YFP/XCR1-KikGR mice in which all DCs express YFP but XCR1^+^ DCs also express KikGR (and can be labeled by photoconversion). This allowed us to use YFP to visualize DC clusters and KikGR to mark XCR1^+^ DCs and identify the location of cDC1s and XCR1^+^ LCs within the DC clusters. Outside of the clusters, we observed single YFP^+^ cells with long dendrites in the epithelium and single KikGR-Red XCR1^+^ DCs with few or no dendrites in the LP (Figure 6B and Supplemental Video 4, KikGR-Red cells in white). Whereas within the YFP^+^ DC clusters, XCR1^+^ DCs were distributed throughout the cluster (Figure 6C and Supplemental Video 5), XCR1^+^ DCs with few or no dendrites (likely cDC1s) were found in the LP, while XCR1^+^ DCs with long dendrites were near the surface (likely XCR1^+^ LCs based on their morphology and location). Furthermore, in some cases XCR1^+^ DCs were localized near the surface (Figure 6D and Supplemental Video 6). These results indicate that DC clusters are composed of a heterogeneous mix of cDC1s and XCR1^+^ LCs.

### DC clusters in the sublingual mucosa contain CD4^+^ Tregs, while most CD8^+^ T cells are found in the epithelium.

**Figure 7:**
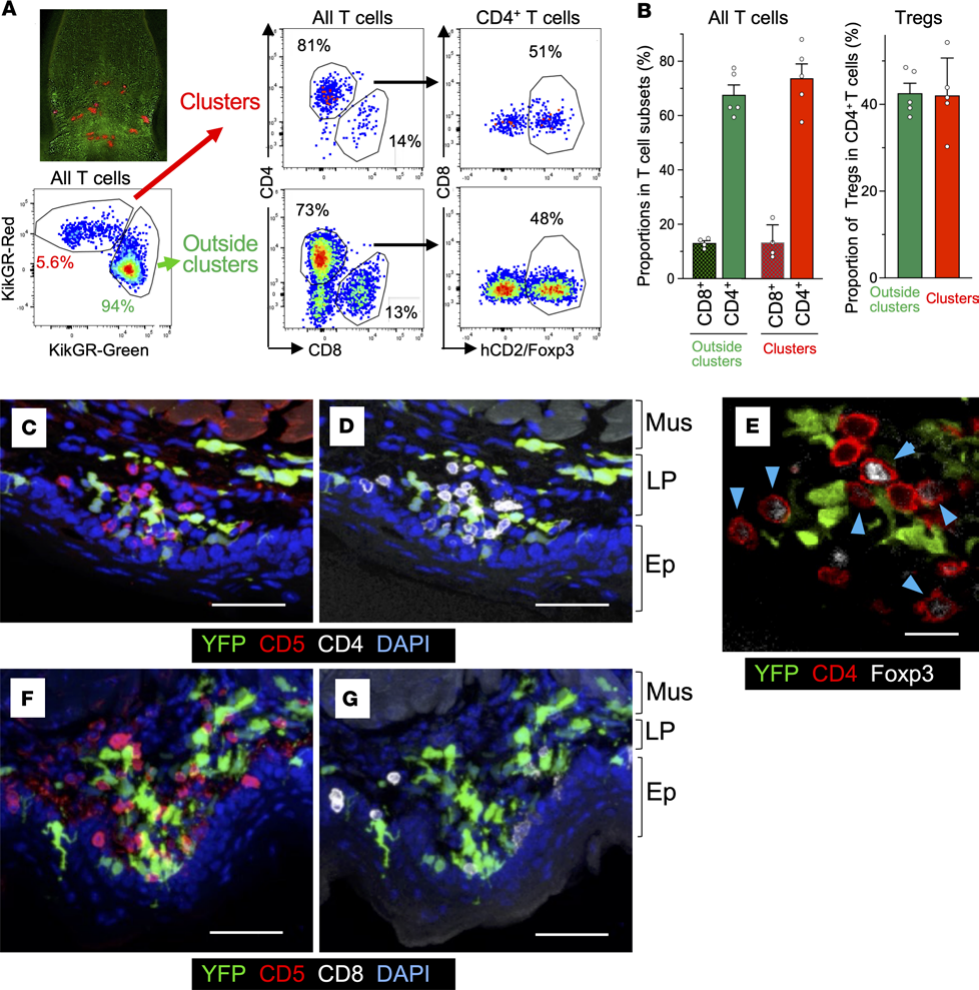
Foxp3^+^ and Foxp3^–^ CD4^+^ T cells and CD8^+^ T cells in sublingual DC clusters. (**A**) Sublingual KikGR^+^ DC clusters in hCD2/CD52-Foxp3/KikGR mouse bone marrow chimeric mice were photolabeled similarly to those in Figure 5A and single-cell suspensions were stained with fluorochrome-conjugated antibodies. Representative flow cytometry plots of photolabeled KikGR-Red T cells in clusters and non-photolabeled T cells outside clusters. (**B**) Proportions of CD4^+^ T cells and CD8^+^ T cells in all T cells (left) and Foxp3^+^ cells in CD4^+^ T cells (right). Data represent mean ± SEM (*n* = 5). (**C**, **D**, **F**, and **G**) Images of a frozen section of sublingual tissue of CD11c-YFP mice, stained with anti-CD5 mAb (red), anti-CD4 mAb (white), and DAPI (blue) (**C** and **D**) (Supplemental Video 7), or stained with anti-CD5 mAb (red), anti-CD8 mAb (white), and DAPI (blue) (**F** and **G**) (Supplemental Video 8). (**E**) Images of a frozen section of sublingual tissue in CD11c-YFP mice stained with anti-CD4 mAb (red) and anti-Foxp3 mAb (white). The data are representative of at least 3 independent experiments. Scale bars: 50 μm (**C**, **D**, **F**, and **G**) and 20 μm (**E**). Mus, muscle layer; LP, lamina propria; Ep, epithelium.

Previous studies reported the presence of T cell–DC clusters in infected and inflamed tissues (8). Our finding that CD11b^+^ DCs and cDC1s are present in the clusters suggests that CD4^+^ and CD8^+^ T cells (7, 12–14, 42) may also be found in these clusters since the 2 DC subsets serve as antigen-presenting cells for these T cells. In addition, oral Tregs are important for maintaining tolerance (4, 5, 9, 15, 19–21). To determine whether T cells, and especially Tregs, are present in the DC clusters, we generated human (h) CD2/CD52-Foxp3/KikGR bone marrow chimeric mice in which KikGR is only expressed in hematopoietic cells and Foxp3^+^ Tregs can be identified as hCD2^+^ cells (43, 44). Using these chimeric mice, we specifically photolabeled KikGR^+^ clusters in the sublingual mucosa and analyzed photoconverted cells by flow cytometry as in Figure 5A and Figure 7A. KikGR-Red^+^ T cells from photoconverted clusters accounted for approximately 5% of total KikGR^+^ T cells. The frequency of CD8^+^ T cells was greater than 10% and similar in and outside of the clusters. CD4^+^ T cells comprised the majority of T cells both in the clusters and outside, and their frequency was slightly higher in the clusters. CD4^–^CD8^–^ T cells were mostly excluded from the clusters (Figure 7, A and B, and Supplemental Figure 8A). These results indicate that both CD4^+^ and CD8^+^ T cells infiltrate DC clusters, but CD4^+^ T cells were the major T subset in the clusters.

Notably, Tregs made up approximately half of the CD4^+^ T cells in the clusters (Figure 7, A and B). The chimerism rate for all T cells was approximately 50%, and this rate varied between T cell subsets (CD8^+^ T cells, 70%; CD4^+^ T cells, 50%; Tregs, 40%). However, a substantial proportion of each T cell subset were KikGR^+^ donor-derived cells (Supplemental Figure 8). Therefore, the relative proportion of each T cell subset within clusters and outside obtained based on KikGR^+^ cells is an accurate reflection of the sublingual T cell subsets. Collectively, these results show that in the steady state T cells infiltrate DC clusters. T cell subsets within the clusters include CD4^+^ T cells with a high frequency of Foxp3^+^ Tregs and CD8^+^ T cells.

Next, we visualized T cells in the DC clusters using CD5, a pan T cell marker (Figure 7C and Supplemental Video 7). Our analysis showed that most T cells (CD5^+^) were CD4^+^ (Figure 7, C and D, and Supplemental Video 7). We also observed that CD4^+^ T cells and DCs were in close contact with each other both in the LP and the epithelium (Figure 7D and Supplemental Video 7). In addition, we observed Foxp3-positive and -negative CD4^+^ T cells in contact with DCs in the clusters (Figure 7E). In contrast with CD4^+^ T cells localized in the LP, most CD8^+^ T cells were in the epithelium in these clusters (Figure 7, F and G, Supplemental Figure 9, and Supplemental Videos 8 and 9). Since DCs and T cells formed dense contacts within the clusters, we named them SLICs.

Tertiary lymphoid structures are found in cancer and chronic inflammatory localizations. They include DCs, T cells, as well as B cells and blood vessels, and are structurally similar to LNs (45, 46). Thus, to investigate the possibility that SLICs resemble tertiary lymphoid structures, we assessed B and IgA^+^ cell number in sublingual immune cells by flow cytometry using targeted in situ photolabeling of KikGR bone marrow chimeric mice (as in Figure 7). However, B cells and IgA^+^ cells were not detected in our samples (data not shown). To confirm this, we generated CD11c-YFP CD19-Cre/CAG-tdTomato flox mice (47, 48) in which DCs express YFP and B lineage cells (including plasma cells) express tdTomato. We observed rare tdTomato-positive cells scattered in the sublingual mucosa, but not in the SLICs (Supplemental Figure 10). These results suggested that SLICs did not have the same immune composition as tertiary lymphoid structures.

Innate lymphoid cells (ILCs) reside in mucosal tissues and ILC2s act as the innate counterparts of Th2 cells by secreting type 2 cytokines (49, 50). Therefore, we investigated whether SLICs contain ILCs using KikGR bone marrow chimeric mice with targeted in situ photolabeling. We confirmed that ILCs (linage^–^CD90^+^ gated) were photoconverted as efficiently as DCs (Figure 5F) and T cells (Figure 7A). However, ILC2 frequency was higher outside the photoconverted SLICs (Supplemental Figure 11). This suggests that SLICs were not considered to be a place where ILC2s specifically accumulate. Altogether, we found that SILCs at least consist of DCs, T cells, and ILCs.

### SLICs contain both CD4^+^ and CD8^+^ T cells and are enriched for CD8^+^ Trms.

**Figure 8:**
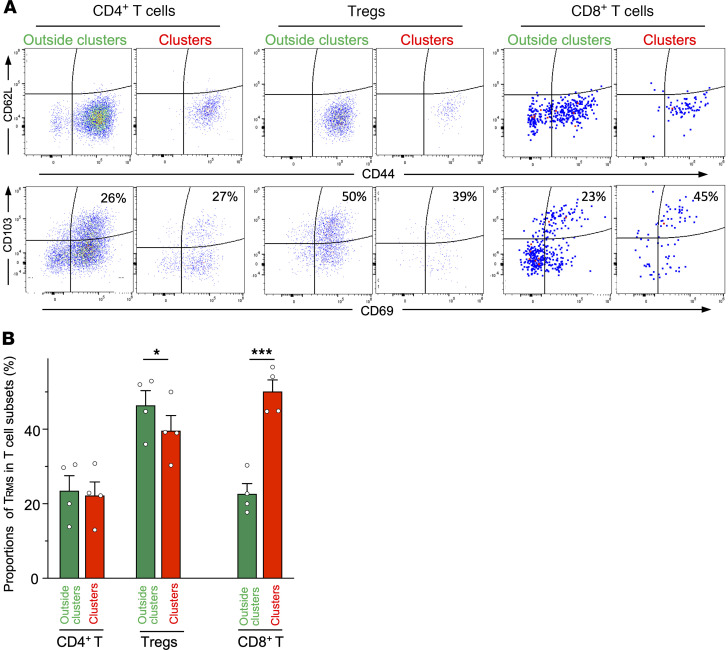
Trm and Treg subsets in the SLICs. Sublingual KikGR^+^ DC clusters in hCD2/CD52-Foxp3/KikGR bone marrow chimeric mice were photolabeled as in Figure 5A and single-cell suspensions were stained with fluorochrome-conjugated antibodies. Representative flow cytometry plots of photolabeled KikGR-Red T cells in clusters and non-photolabeled T cells outside clusters. (**A**) Representative flow cytometry plots of T cells. (**B**) Proportions of Trm cells in each T cell subset. Data represent mean ± SEM (*n* = 4). Statistical comparisons were performed using an unpaired, 2-tailed Student’s *t* test. *P < 0.05, ***P < 0.001.

Immune cell clusters in peripheral tissues are involved in Trm maintenance and functions (8). Therefore, we investigated the properties of T cells, and specifically Trms, within SLICs. Almost all CD4^+^ and CD8^+^ T cells were CD62L^–^CD44^med/hi^ memory/effector phenotype both within and outside of the SLICs. There was no difference in the proportion of CD4^+^CD69^+^CD103^+^ Trms within the SLICs compared to outside, but the proportion of Tregs was lower in the SLICs. In contrast, the proportion of CD8^+^CD69^+^CD103^+^ Trms was increased 2-fold within the SLICs (Figure 8).

### SLIC response to inflammation.

**Figure 9:**
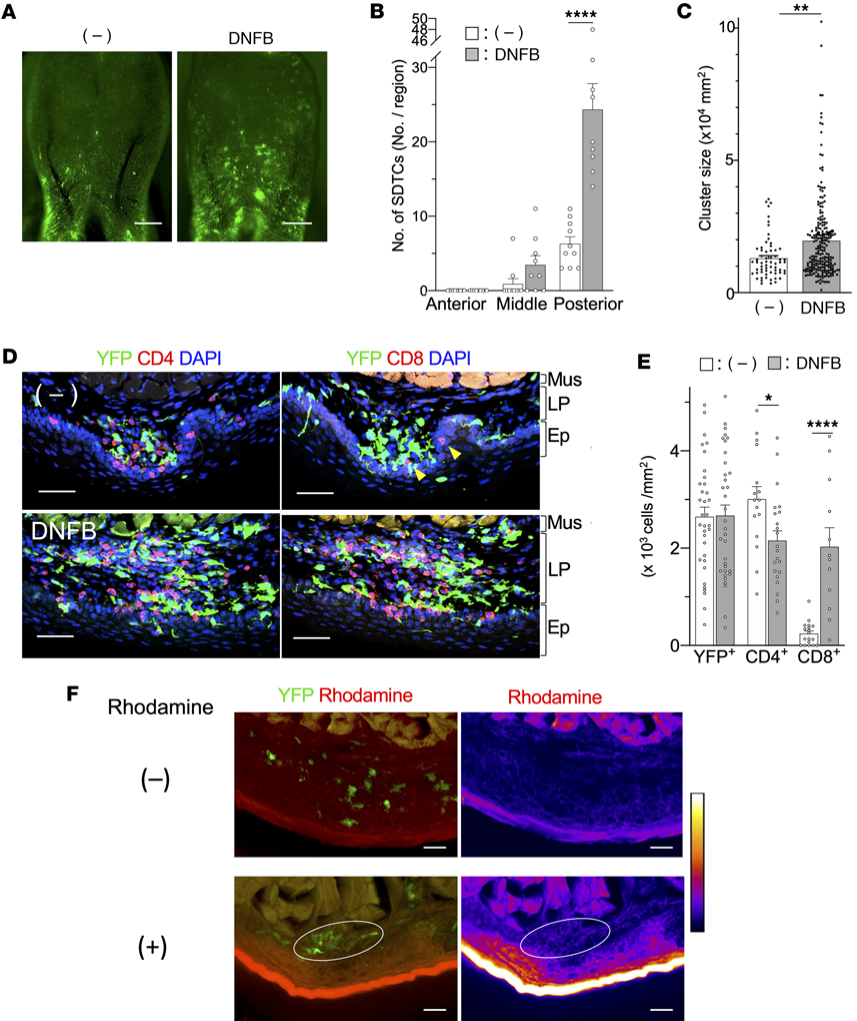
Inflammation increases the number and size of SLICs. DNFB was applied to the sublingual mucosa of CD11c-YFP mice. (**A**) Image of sublingual mucosa before application (left panel) and 1 day after the second application (right panel). Scale bars: 1 mm. (**B**) Numbers of SLICs before and after application of DNFB. Fluorescence images of sublingual region shown in **A** were subdivided into the anterior (red square), middle (light-blue square), and posterior (blue square) regions as in Figure 4B and number of SLICs was counted. (**C**) Size of SLICs in posterior sublingual region before and after application of DNFB. (**D**) Images of serial frozen sections of sublingual tissue of CD11c-YFP mouse before (upper panels) and after application of DNFB (lower panels). Serial tissue sections were stained with anti-CD4 mAb or anti-CD8 mAb (red) and DAPI (blue) (Supplemental Video 10). Yellow arrowheads in upper right panel point to CD8^+^ cells. Scale bars: 50 μm. (**E**) Densities of DCs, CD4^+^ T cells, and CD8^+^ T cells in SLICs. Data in **B**, **C**, and **E** represent mean ± SEM. At least 9 samples (**B** and **C**) and 12 samples (**E**) from in each group were analyzed. (**F**) Fluorescence images of YFP and rhodamine signals (left panels) and rhodamine signal (right panels) of inflamed sublingual region 20 minutes after rhodamine application. White circles indicate SLIC. Scale bars: 50 μm. The data are representative of at least 3 independent experiments. Statistical comparisons were performed using an unpaired, 2-tailed Student’s *t* test. *P < 0.05, **P < 0.01, ****P < 0.0001. Mus, muscle layer; LP, lamina propria; Ep, epithelium.

Immune cell clusters formed by inflammation and infection in the skin and vagina play an important role in the immune response in the barrier tissues (24–26). To investigate how SLICs responded to inflammation, 2,4-dinitrofluorobenzene (DNFB) was applied twice (on days 0 and 5) to the sublingual region of CD11c-YFP mice (51) and the area was visualized 24 hours later. We observed that the number of SLICs in the posterior sublingual region increased from 6.3 to 24.3 in response to inflammation (Figure 9, A and B). After DNFB treatment, we found some SLICs that were similar in size to the steady-state SLICs as well as some much larger SLICs. On average, SLIC area increased from 1.32 × 10^4^ to 1.96 × 10^4^ μm^2^ (Figure 9C) in the posterior sublingual region.

Next, we visualized DCs and T cells and measured their densities in the SLICs (Figure 9, D and E). During inflammation, SLICs extended from the LP to epithelium. While CD4^+^ T cells were the major T cell subset in the SLICs in the steady state (Figure 7 and Figure 9D), following DNFB treatment, the number of CD8^+^ T cells substantially increased and they predominantly localized to the LP (Figure 9D, Figure 9E, and Supplemental Video 11). DCs and CD4^+^ and CD8^+^ T cells were in close contact with each other. DC density remained unchanged in response to inflammation, while the density of CD4^+^ T cells decreased slightly. In contrast, CD8^+^ T cell density increased substantially and was similar to CD4^+^ T cell density following DNFB treatment (Figure 9E). Furthermore, to investigate whether foreign substances in the oral cavity come into contact with SLICs, we applied the fluorescent dye rhodamine to the inflamed sublingual mucosa and found that it penetrated to the area where SLICs were present (Figure 9F). Thus, foreign substances penetrate into SLICs during inflammation and this may induce more rapid immune responses. These results suggest that SLICs form a unique microenvironment that facilitates T cell–DC interactions during inflammation and mediates the oral immune response.

## Discussion

Here, we combined tissue clearing with in situ photolabeling to investigate the distribution of DCs throughout the mucosa of the oral cavity. We found that DCs were nonuniformly distributed and detected concentrated regions or clusters of multiple DCs in close proximity to each other. Specifically, we found clusters (SLICs) containing T cells in close contact with DCs that were located primarily in the posterior region and extended from the LP to the epithelium of the sublingual mucosa. Most DCs in the SLICs were CD11b^+^ DCs (with some cDC1s), while the T cell population in the clusters was composed of a large proportion of CD4^+^ T cells, particularly Tregs, and some CD8^+^ T cells. The T cells were of a memory/effector phenotype and included Trms. Our results demonstrate the existence of specialized immune foci in the oral cavity that facilitate DC–T cell interactions in steady state and inflammation.

SLICs differ from the clusters formed around hair follicles in intact uninjured skin (23) since SLICs have a relatively high Treg content (around 50% of all CD4^+^ T cells). The SLICs that we identified are also distinct from iSALT observed in a contact hypersensitivity model (24), since the latter structures were only detected during inflammation but the SLICs are found in the steady state. It has been shown that Foxp3^+^ Tregs are highly enriched in the oral mucosa, particularly in the LP of the posterior tongue compared with the small intestine or secondary lymphoid organs (52). These Tregs are distinct from LN or spleen Tregs, as they express high levels of the tissue retention molecule CD103 as well as CTLA4 (52). These findings suggest that Tregs in SLICs may possess a phenotype specific to the oral mucosa. Collectively, these reports and our findings point to an important role for Tregs within SLICs in maintaining oral homeostasis, including tolerance to environmental antigens, such as food antigens and commensal bacteria in the oral cavity. Since SLICs are enriched in mucosal Tregs, they may facilitate effective allergen SLIT (4, 5). On the other hand, oral equilibrium in response to *Candida albicans*, a common commensal microbe, and protection against acute infections are mediated by IL-17–producing γδ T cells and Th17-type Trm cells (53, 54). Furthermore, we found that almost half of CD4^+^ T cells in the SLICs were non-Foxp3^+^ Tregs. Therefore, it is possible that memory Th17, as well as other CD4^+^ T cell subsets, are modulated by DCs within the SLICs.

Our analysis revealed that DCs persist in the vestibular region and some areas of the mandibular region, such as the posterior side of the sublingual region, gingiva, and buccal mucosa. Since these regions are prone to physical stimulation from chewing, food residue, and saliva, it is possible that the frequent stimulation leads to a localized increase in DC density. The vestibule is considered to be a suitable site for SLIT administration because of the high number of LCs in this region (55). Our results confirmed that the oral vestibule did indeed have more DCs. However, we also showed that DCs were nonuniformly distributed within each region and it is the posterior sublingual region that had a high density of DCs. We cannot rule out the oral vestibule as a site of allergen immunotherapy administration based on DC distribution, but we believe that the sublingual region with its high DC density is another potential site for administration of allergen immunotherapy. We also demonstrated that CD11b^+^CD11c^+^ DCs are the major DC subset in the oral cavity, both within and outside of SLICs. This subset is thought to be key for mediating oral tolerance by stimulating Treg induction in the dLNs through retinoic acid and TGF-β production (15, 21) (in addition to the sublingual CD11b^+^CD11c^–^ macrophage-like interstitial cells; ref. 19). Taken together, these studies suggest that the sublingual route is a valuable site for administration of SLIT.

Recent reports suggest that CD103^+^ LCs account for nearly half of LCs in the buccal and gingival mucosa, and that 10% to 20% of these LCs express the cDC1 marker XCR1 (10). However, unlike LCs in skin, these CD103^+^ LCs are unable to present antigen to CD8^+^ T cells and are not involved in CD8^+^ T cell activation (10, 56). In our study, we found that almost 20% of LCs in the sublingual mucosa were XCR1^+^CD103^+^ LCs and most XCR1^+^ DCs with long dendrites (also likely to be LCs) were found in the epithelium. Furthermore, CD8^+^ T cells were also abundant in the same region. Therefore, we hypothesize that XCR1^+^ LCs (together with cCD1s) may play a role in the maintenance of CD8^+^ Trms specific for commensal microorganisms or food antigens in SLICs in the LP and epithelium in the steady state. Future studies will elucidate the exact functions of CD103^+^XCR1^+^ LCs in the clusters.

Earlier studies indicate that the proportion of CD4^+^ T cells in the oral mucosa is higher than that of CD8^+^ T cells (52). Consistent with these reports, we found a greater proportion of CD4^+^ T cells compared with CD8^+^ T cells both within and outside the SLICs.

Additionally, it has been shown that T cells have a memory/effector phenotype and CD4^+^ Trms are present in mixed oral mucosa, but not in the spleen (57). Reports show that CD4^+^ Trm cells are located in the dermis or LP, while CD8^+^ Trm cells express CXCR9 and CXCR10 and are located in the epidermis or epithelium during infection in the skin and mucosa (8). We showed that CD4^+^ T cells were present in the LP, while CD8^+^ T cells were in the epithelium in the SLICs. In other words, the localization patterns of CD4^+^ Trm cells and CD8^+^ Trm cells in these tissues are similar to the SLICs. It is likely that SLICs support both CD4^+^ and CD8^+^ Trms similarly to immune cell clusters in other tissues. Notably, a higher frequency of CD8^+^ Trms and XCR1^+^ cDC1s in the SLICS suggests that SLICs provide a unique microenvironment for maintenance and function of CD8^+^ Trms.

The formation of SLICs is relevant to inoculation of food antigens, changes in bacterial flora due to diet, and physical stimulation of the sublingual mucosa by food and other substances, even under specific pathogen–free conditions. The differences in antigen specificity of T cells (including Trms), inside and outside the SLIC, as well as cytokine production and molecular expression, are important for determining the role of SLICs and the mechanism of their generation, which will be clarified in future single-cell analyses.

An earlier study showed that in a contact hypersensitivity model where DNFB was applied to the abdominal skin and then to the buccal mucosa, the proportion of CD8^+^ T cells in the buccal mucosa increased from 20% to 60% after DNFB treatment (58). Likewise, in our study, DNFB application markedly increased the density of CD8^+^ T cells to the same level as CD4^+^ T cells in the SLICs. In addition, we observed that exogenous substances could penetrate to the SLICs. This suggests that CD8^+^ T cell accumulation is a common feature of oral mucosal immune responses and that SLICs may mediate inflammatory immune responses to haptens by providing a unique microenvironment that regulates CD8^+^ T cell immune responses.

Taken together, our results suggest that the SLICs we identified in the oral cavity act as local immunoregulatory sites by bringing DCs into close proximity to CD4^+^ and CD8^+^ effector/memory T cells and Treg cells and facilitating their interactions. Improved understanding of the generation and function of SLICs, particularly during antigenic stimulation, will reveal the mechanisms of local secondary immune regulation in the oral cavity and facilitate further development of needle-free vaccines and SLIT.

## Methods

### Sex as a biologic variable.

Sex was not considered as a biological variable. Both male and female mice were included in this study.

### Mice.

CD11c-YFP mice were provided by Michel C. Nussenzweig (The Rockefeller University, New York, New York, USA) (30). XCR1-KikGR mice (41) used in this study were generated in-house. hCD2/CD52-Foxp3/KikGR mice were generated by mating KikGR mice (59) and hCD2/CD52-Foxp3 mice (43). Langerin-Cre/KikGR mice were generated by mating Langerin-Cre mice (60) with ROSA-CAG-Loxp-stop-Loxp-KikGR mice (59). CD11c-KikGR mice were generated by transfecting a construct composed of KikGR-cDNA downstream of the CD11c promoter (gift from Michel C. Nussenzweig) into embryonic fertilized eggs of C57BL/6 mice. CD11c-YFP CD19-Cre/CAG-tdTomato flox mice were generated by mating CD19-Cre mice (47), CAG-Loxp-stop-Loxp-tdTomato mice (61), and CD11c-YFP mice.

### Preparation of tissue-cleared samples.

Tissue clearing of tongue or buccal mucosal tissues was performed using the Sca*l*eS method (31). Resected tongue or buccal mucosal tissues was fixed with 4% paraformaldehyde/PBS (PFA/PBS) (w/v) at 4°C for 72 hours and then treated with Sca*l*e SQ (5) solution at 37°C for 14 days. For observation of buccal mucosa, the surface of the tissues was stained using 20 nM Cell Tracker Orange (Invitrogen) diluted in Sca*l*e-S4 solution at room temperature for 15 minutes. DNA was stained with 300 nM 4′,6-diamidino-2-phenylindole (DAPI, Molecular Probes) or 100 ng/mL propidium iodide (PI) in S4 solution at room temperature for 3 days.

### Photoconversion, cell isolation, and flow cytometric analysis of KikGR^+^ cells in the sublingual clusters.

The clusters composed of cells derived from hCD2/CD52-Foxp3/KikGR mouse bone marrow cells on the sublingual surface of resected tongue were irradiated with violet laser light utilizing the ROI function by confocal laser scanning microscopy (A1, Nikon). Cells of sublingual mucosa (only bottom surface of tongue without oral floor) were enzymatically isolated by Liberase TL (Roche Diagnostics) according to the method reported by Park et al. (57). Briefly, the tissue was cut into small pieces and treated with Liberase TL (0.5 mg/mL) for 1.5 hours at 37°C. The enzyme reaction was stopped by EDTA (1 mM). From the recovered cells, the CD45^+^ cells were sorted with anti-CD45 MACS bead (Miltenyi Biotec). Sorted CD45^+^ cells were stained with fluorochrome-conjugated mAbs shown in Supplemental Table 2 and analyzed by flow cytometry (SP6800, SONY). KikGR^+^ clusters in CD11c-KikGR mice were photolabeled and analyzed in the same way as above. Acquired flow cytometric data were analyzed by FlowJo (Tree Star).

### Generation of bone marrow chimeric mice.

The recipient C57BL/6 male mice (6–10 weeks old) were irradiated with 10.5–11 Gy of x-rays (mediXtec). After 2 to 3 hours, 2 × 10^6^ to 4 × 10^6^ bone marrow cells obtained from sex-matched hCD2/CD52-Foxp3/KikGR mice (6–10 weeks old) were transplanted into the irradiated recipient mice. Two months later, chimeric ratios of B cells in blood were confirmed to be above 90%.

### Application of DNFB to the sublingual region.

Ten microliters of 0.5% and 0.3% DNFB (Nacalai Tesque) dissolved in acetone/olive oil (both Wako) (4:1) was painted on the sublingual mucosa of the CD11c-YFP mice on days 0 and 5, respectively. On day 6, the tongue was resected and observed by confocal microscopy (LSM 510 META, Zeiss or A1, Nikon) or fixed for immunohistochemical analysis.

### Immunohistochemistry.

Ten- or 20-μm frozen sections of tongue obtained from CD11c-YFP mice were prepared using a cryostat (Leica CM1850) and fixed with cold acetone for 5 minutes as previously described (62). After blocking with 3% BSA in PBS, the tissue sections were stained with allophycocyanin-conjugated (APC-conjugated) rat anti–mouse CD45 mAb (30-F11, eBioscience), APC- or phycoerythrin-conjugated (PE-conjugated) rat anti–mouse CD5 mAb (53-7.3, BioLegend), Alexa Fluor 647–conjugated anti–mouse CD4 mAb (GK1.5, BioLegend), APC-conjugated anti–mouse CD8α mAb (53-6.7, BioLegend), or Alexa Fluor 647–conjugated anti–mouse Foxp3 mAb (MF-14, BioLegend), and DAPI. For assessment of rhodamine penetration into sublingual tissue, 1 day following the second application of DNFB, rhodamine (2 ng/mL, 10 μL) was applied to the sublingual mucosal surface. Twenty minutes later, the tongue was removed, fixed in PFA, cryoprotected in 30% sucrose, and embedded in OCT. Cryosections were prepared and visualized using a confocal laser microscope.

### Observations and image processing.

Intact oral tissues and sections subjected to immunohistochemistry were observed using a fluorescence microscope (Nikon) utilizing tiling or *Z*-stack imaging. The *Z*-stack images of tissue-cleared samples were obtained using a confocal microscope (LSM 510 META, Zeiss or A1, Nikon) or light-sheet microscope (Light Sheet Z.1, Zeiss). Extended depth of focus (EDF), 3D, or orthogonal reconstruction (Zen lite, Zeiss or NIS-Elements, Nikon) was applied to the *Z*-stack images. To observe deeper regions of the dorsal or sublingual tongue, the cut surface of a coronal section of the tongue was observed using fluorescence or confocal microscopy. A DC cluster was defined as 7 or more DCs in contact with each other. In cases when overlapping DCs were present and the number of cells could not be determined, a diameter of more than 30 μm was defined as a DC cluster. To quantitate the numbers of single DCs or clusters, the *Z*-stack images of the sublingual region were acquired. Maximal projections of *Z*-stack images were generated using EDF processing. The sublingual region was subdivided into 3 regions, and the numbers of single and clustered DCs in each region were manually scored as YFP^+^ and PI^+^ cells. Quantitation of the number of DC clusters was performed separately by 5 investigators, and the average number of DC clusters/mm^2^ in each region was calculated.

### The measurement of SLIC area and density analysis for DCs and CD4^+^ and CD8^+^ T cells in SLICs.

Tongues of resting and DNFB-treated CD11c-YFP mice were excised, and *Z*-stack images of the sublingual surface were acquired in 4-μm steps by confocal laser microscope (Nikon). For each SLIC, the section of the *Z*-image with the largest size of that SLIC was selected. Then, the area (μm^2^) of each SLIC was measured by enclosing the SLIC manually by ROI, using the edge of the YFP^+^ cells as an indicator of the edge of the SLIC (NIS-Elements, Nikon). For the measurement of densities of DCs, CD4^+^ cells, and CD8^+^ cells in SLICs, 10-μm-thick serial frozen tissue sections of the steady-state and DNFB-treated tongues of CD11c-YFP mice were stained with DAPI and APC- or Alexa Fluor 647–conjugated anti–mouse CD4 mAb or APC-conjugated anti–mouse CD8α mAb and images acquired by fluorescence microscopy (Nikon). The area (μm^2^) of each SLIC was measured by enclosing the SLIC manually by ROI, using the YFP^+^ cells at the edge of the SLIC as an indicator. The numbers of each cell type in an SLIC were counted, confirming the presence of nuclei by DAPI staining, and cell densities within each SLIC were calculated by dividing the number of cells by the area (μm^2^).

### Statistics.

One-way ANOVA with Tukey’s multiple-comparison test or the unpaired Student’s *t* test was conducted using Prism (GraphPad Software). Data are presented as the mean ± SEM. A *P* value of less than 0.05 was considered to indicate a significant difference.

### Study approval.

These mice were bred in specific pathogen–free animal rooms at Osaka Ohtani University. All experimental procedures were approved by the Institutional Animal Care and Use Committee of Osaka Ohtani University Faculty of Pharmacy.

### Data availability.

Values for graphs in the figures and Supplemental figures are provided in the Supporting Data Values file.

## Author contributions

Y Kusumoto and MT designed experiments. MU, Y Kusumoto, and MT performed experiments, analyzed data, and performed data visualization. MH, HT, NO, JY, AN, AF, AK, MS, YI, K Okamura, K Obazaki, RK, NS, YT, and Y Kamiya performed experiments. RI and TM analyzed data. T Kaisho, HH, JK, MI, T Honda, and K Kabashima provided the materials. T Hoshida, TK, K Kataoka, TA, TW, and AM technically supported experiments and data validation. MT, Y Kusumoto, and TC wrote the manuscript. All authors reviewed, commented on draft and approved the final manuscript.

## Supplementary Material

Supplemental data

Supplemental video 1

Supplemental video 2

Supplemental video 3

Supplemental video 4

Supplemental video 5

Supplemental video 6

Supplemental video 7

Supplemental video 8

Supplemental video 9

Supplemental video 10

Supporting data values

## Figures and Tables

**Table 1:**
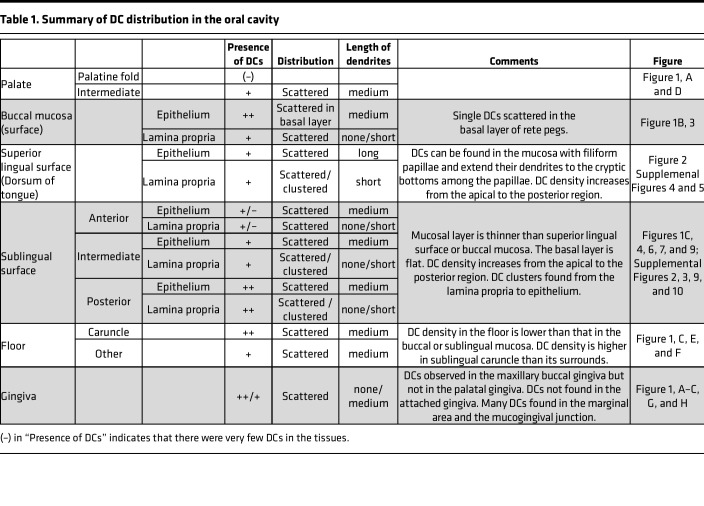
Summary of DC distribution in the oral cavity

## Version 1 10/01/2024

In-Press Preview

## Version 2 11/08/2024

Electronic publication
