# Supplementary material for: Sublingual immune cell clusters and dendritic cell distribution in the oral cavity
Source: JCI Insight. 2024 Oct 1;9(21):e167373. doi: 10.1172/jci.insight.167373 (PMC11601585; doi:10.1172/jci.insight.167373)
Supplement: Supplemental data [file jciinsight-9-167373-s194.pdf]

Supplemental Information for

Sublingual immune cell clusters and dendritic cell distribution in the oral cavity

Supplemental Figure 1. Expression of YFP, CD11c, and MHC class II in sublingual cells.

Supplemental Figure 2. Detection of YFP^{+} cells in the sublingual region of CD11c-YFP mice.

Supplemental Figure 3. Fluorescence images of sublingual tongue in CD11c-YFP mouse and wild type mouse.

Supplemental Figure 4. Differential distribution of DCs in the oral cavity of CD11c-YFP mice. Related to Figure 1.

Supplemental Figure 5. $CD11c^{+}$ cells in anterior region in dorsal surface of tongue. Related to Figure 2.

Supplemental Figure 6. Numbers of DC clusters in sublingual posterior regions at each week of age. Related to Figure 4.

Supplemental Figure 7. Gating strategy for sublingual DCs in CD11c-KikGR mice. Related to Figure 5.

Supplemental Figure 8. Gating strategy for flow cytometric analysis and chimerism rates of sublingual T cells in hCD2/CD52-Foxp3/KikGR mouse bone marrow chimera mice. Related to Figure 7 and 8.

Supplemental Figure 9. Most $CD8^{+}$ T cells are located in epithelium in sublingual DC clusters. Related to Figure 7F, G.

Supplemental Figure 10. Image of B cell-lineage cells in sublingual mucosa.

Supplemental Figure 11. Flow cytometric analysis of ILCs in SLICs in sublingual tissue.

Supplemental Table 1: DC subsets and functions in human and mouse

Supplemental Table 2: Antibodies for Flow cytometry.

Supplemental Information for

Sublingual immune cell clusters and dendritic cell distribution in the oral cavity

Supplemental Movie 1. DCs in the mucosa of dorsal tongue of CD11c-YFP mouse. Related to Figure 2A and Supplemental Figure 5.

Supplemental Movie 2. LCs in the sublingual epithelium with significantly longer dendrites. Related to Figure 4G.

Supplemental Movie 3. DC cluster in the sublingual mucosa comprised DCs with short dendrites. Related to Figure 4I.

Supplemental Movie 4. Scattered $XCR1^{+}$KikGR cells and YFP^{+} cells in the sublingual mucosa of CD11c-YFP/XCR1-KikGR mouse. Related to Figure 6B.

Supplemental Movie 5. The distribution of $XCR1^{+}$ cells and YFP^{+} cells in the sublingual DC cluster of a CD11c-YFP/XCR1-KikGR mouse. Related to Figure 6C.

Supplemental Movie 6. The distribution of $XCR1^{+}$ cells and YFP^{+} cells in the sublingual DC cluster of a CD11c-YFP/XCR1-KikGR mouse. Related to Figure 6D.

Supplemental Movie 7. Immunohistostaining of $CD4^{+}$ cells and $CD5^{+}$ cells in the sublingual DC cluster of CD11c-YFP mouse. Related to Figure 7C, D.

Supplemental Movie 8. Immunohistostaining of $CD8^{+}$ cells and $CD5^{+}$ cells in the sublingual DC cluster of a CD11c-YFP mouse. Related to Figure 7F, G.

Supplemental Movie 9. Immunohistostaining of $CD8^{+}$ cells and $CD5^{+}$ cells in the sublingual DC cluster of CD11c-YFP mouse. Related to Supplemental Figure 9.

Supplemental Movie 10. Immunofluorescence staining of $CD4^{+}$ cells and $CD8^{+}$ cells in the immune cell cluster of CD11c-YFP mouse tongue after DNFB application. Related to Figure 9D.

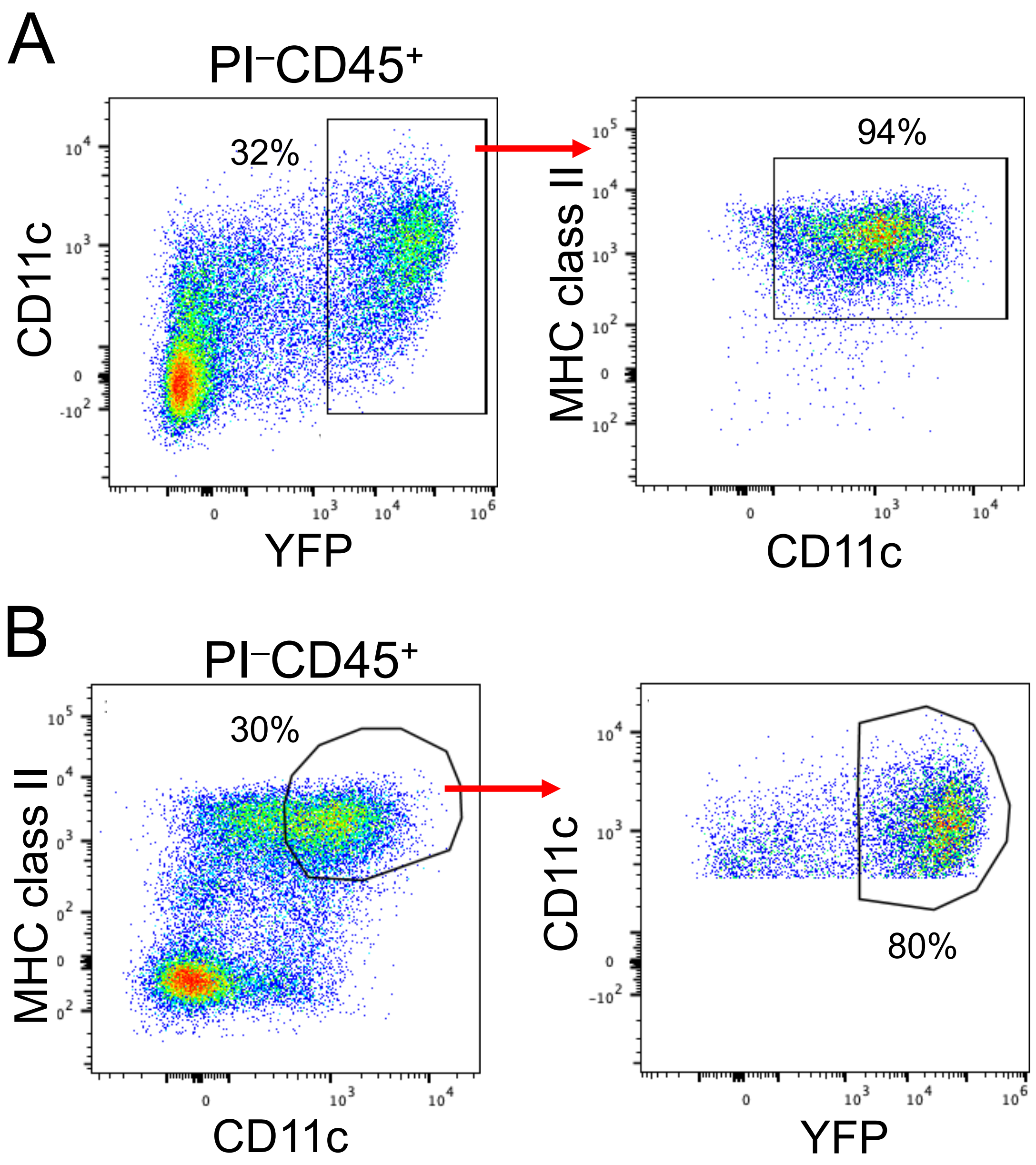

Supplemental Figure 1. Expression of YFP, CD11c, and MHC class II in sublingual cells. Single-cell suspensions prepared from the sublingual region were stained with Phycoerythrin/Cy5-conjugated anti-CD45, Brilliant Violet 410-conjugated anti-CD11c, and Brilliant Violet 510 conjugated anti-MHC class II mAbs. (**A**) YFP^+ cells in sublingual cells express CD11c and MHC class II. (**B**) Expression of YFP in $CD11c^+$MHC class II^+ sublingual cells. The numbers indicate the frequencies of the gated population of parent cells. The data are representative of at least three independent experiments.

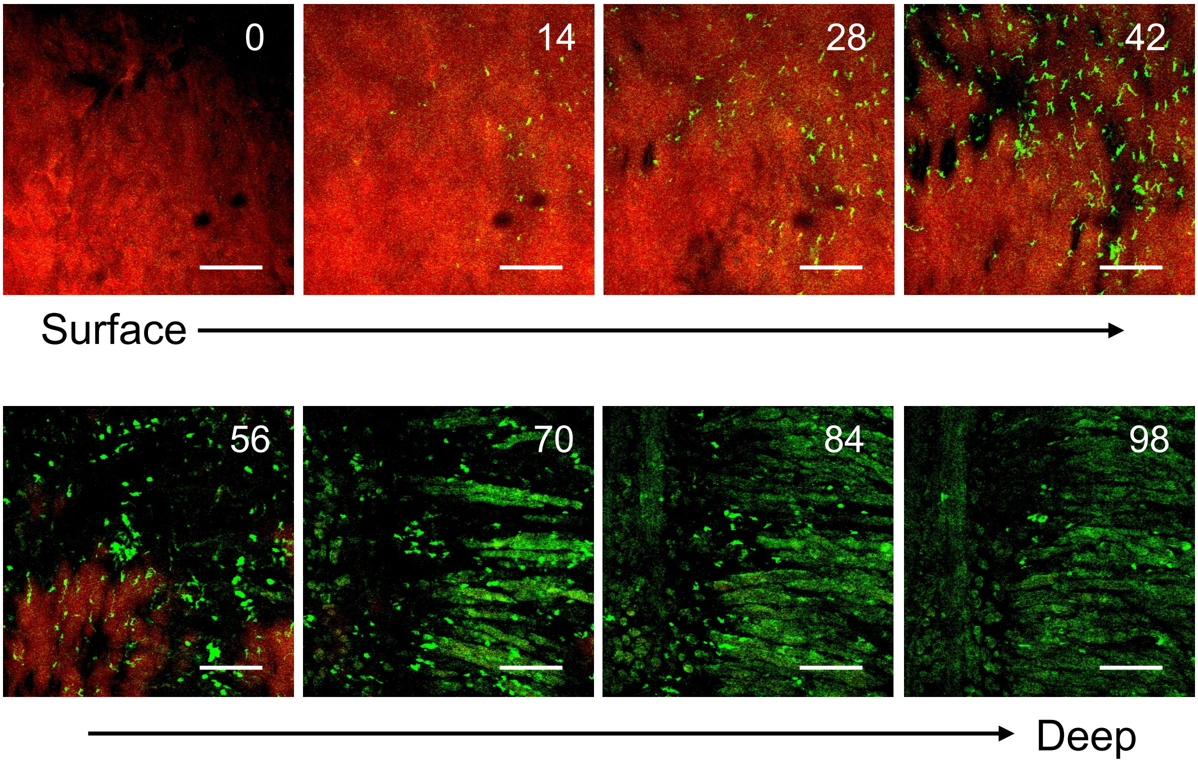

Supplemental Figure 2. Detection of YFP⁺ cells in the sublingual region of CD11c-YFP mice. CD11c⁺ cells in the sublingual mucosa were observed using fluorescence stereoscopic microscopy. z-stack images from the mucosal surface to muscle layer. CD11c-YFP (green), autofluorescence (red). Numbers in images indicate depth (μm) from the surface. The data are representative of at least three independent experiments. Scale bars = 100 μm.

CD11c-YFP mouse   Wild type mouse

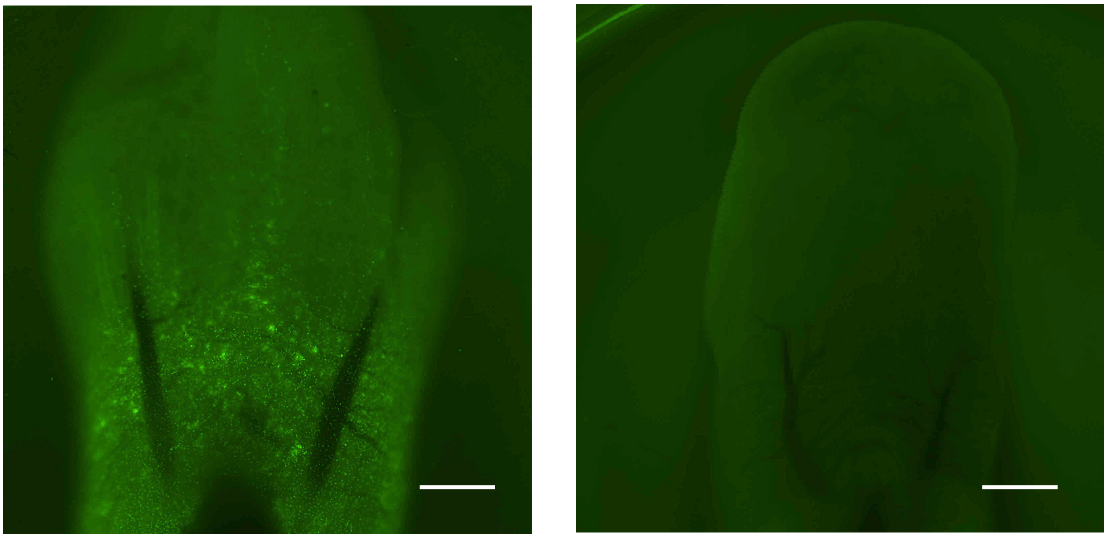

Supplemental Figure 3. Fluorescence images of sublingual tongue in CD11c-YFP mouse and wild type mouse.
Fluorescence signal of sublingual region of CD11c-YFP mouse (left panel) and wild type mouse (right panel) acquired by the same setting via GFP channel by fluorescence stereoscopic microscopy. The data are representative of at least three independent experiments. Scale bars = 1 mm.

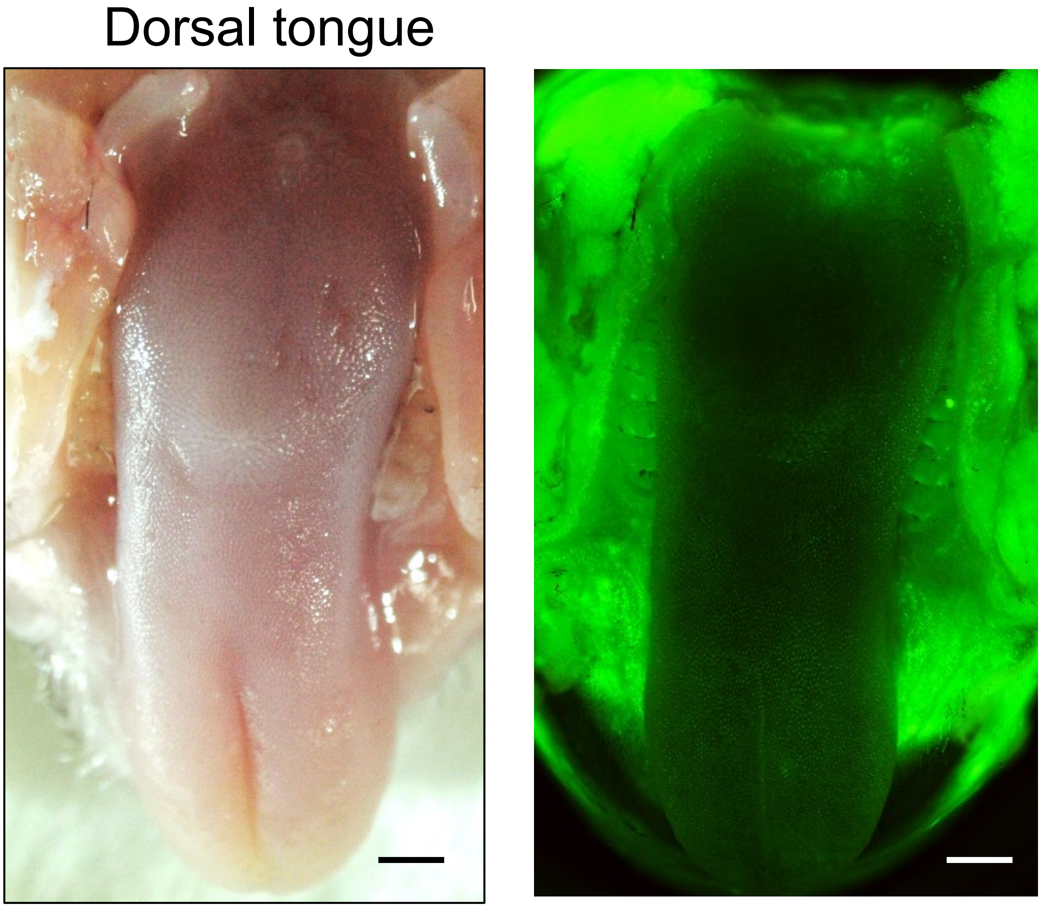

Supplemental Figure 4. Differential distribution of DCs in the oral cavity of CD11c-YFP mice. Related to Figure 1.

Bright-field image (left panels) and fluorescence image (right panel) acquired using fluorescence stereoscopic microscopy. Dorsal mucosa of the tongue. The data are representative of at least three independent experiments. Scale bars = 1 mm.

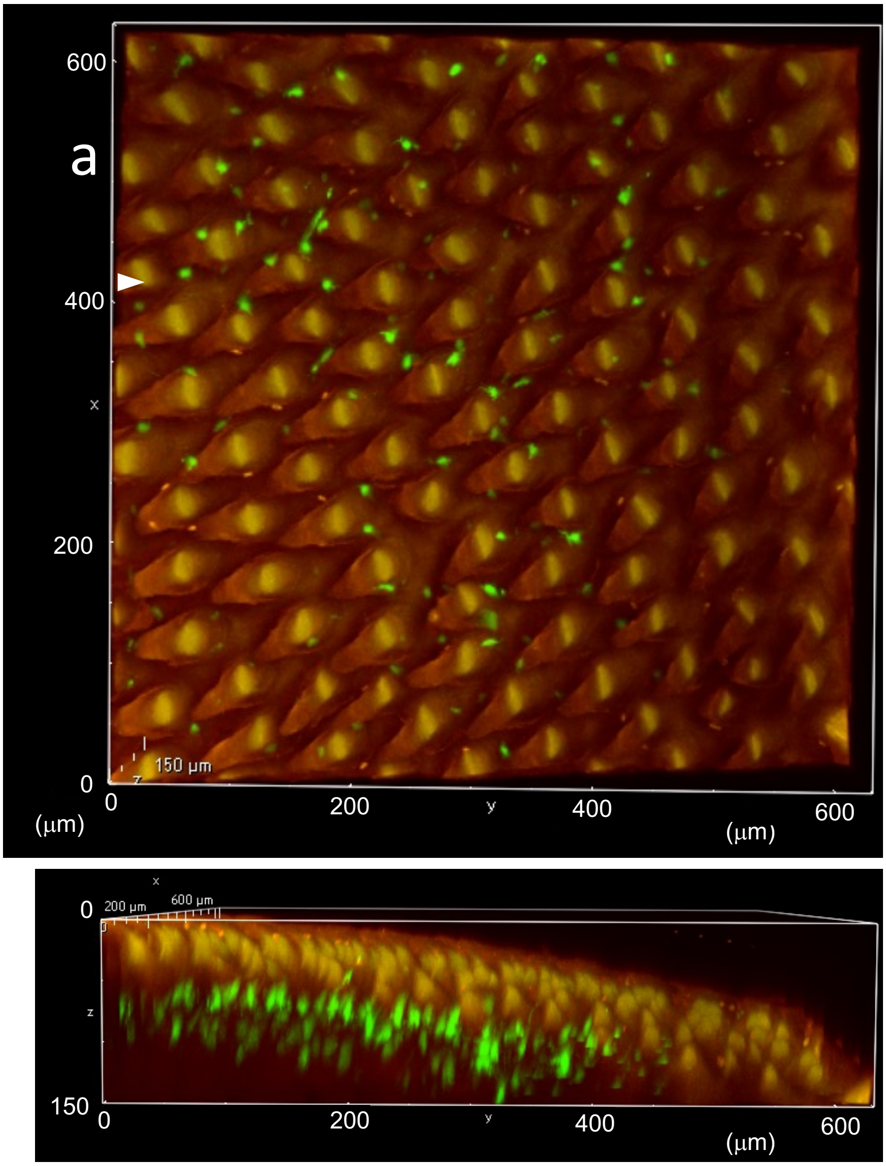

Supplemental Figure 5. CD11c$^+$ cells in anterior region in dorsal surface of tongue. Related to Figure 2. Fluorescence images of DCs in the anterior region of dorsal surface of tongue in CD11c-YFP mice. Vertical image (upper panel) and sagittal image (lower panel) of cleared tongue (Supplemental Movie 1). The data are representative of at least three independent experiments.

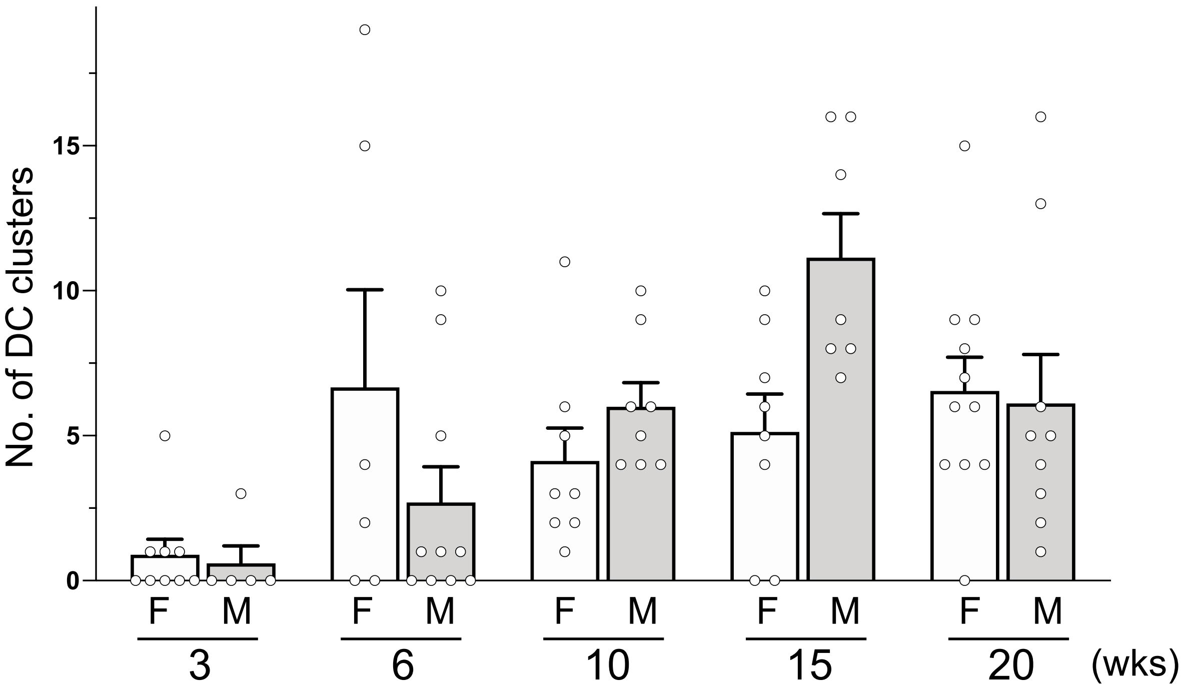

Supplemental Figure 6. Numbers of DC clusters in sublingual posterior regions at each week of age. Related to Figure 4.

Number of DC clusters in posterior regions at weeks of age were counted.
Data represent mean ± SE (n >= 5). F: female, M: male.

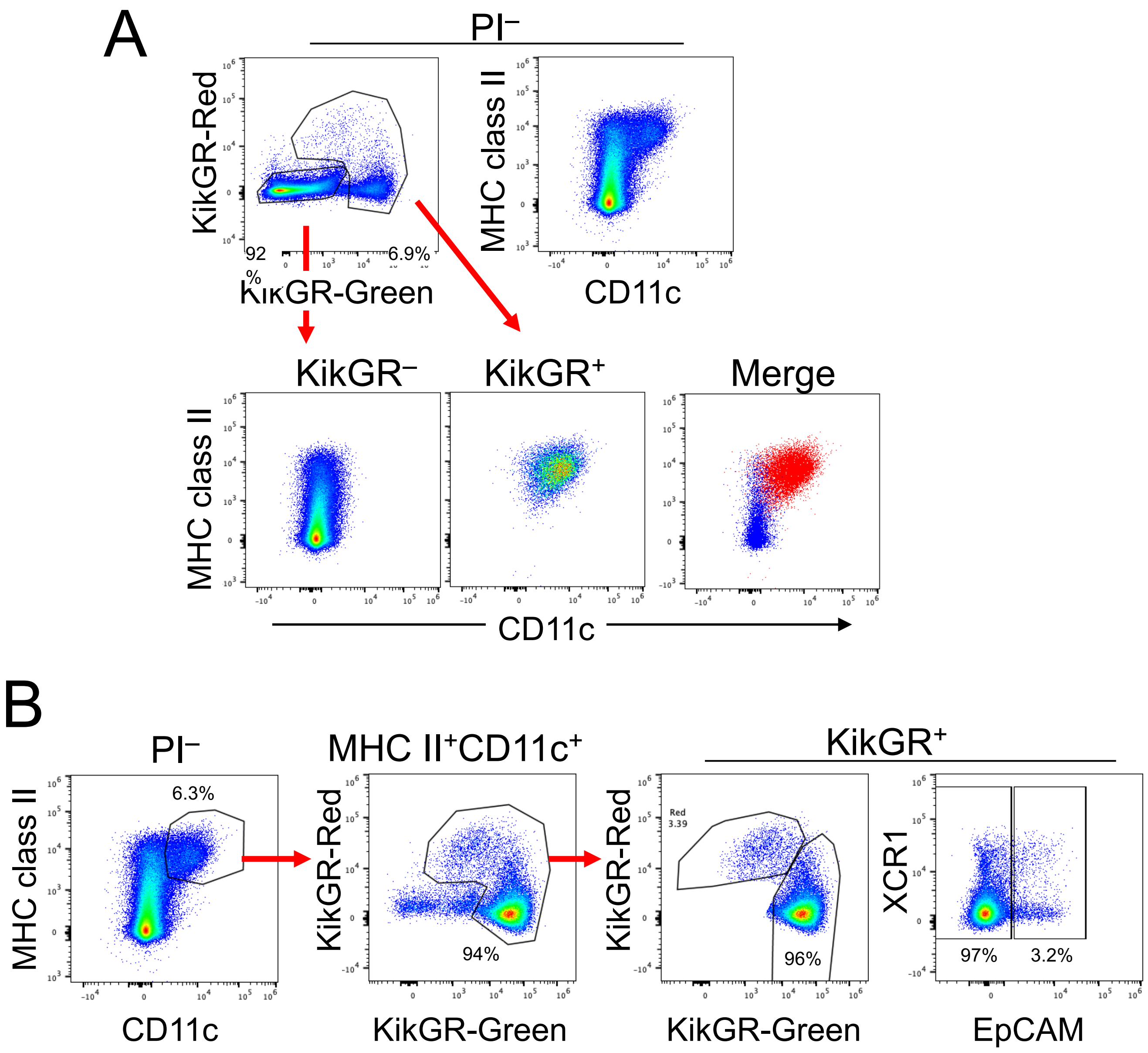

Supplemental Figure 7. Gating strategy for sublingual DCs in CD11c-KikGR mice. Related to Figure 5. Gating strategy of flow cytometric analysis of sublingual cells in CD11c-KikGR mice. (**A**) CD11c^{+} cells specifically express KikGR in CD11c-KikGR mice. (**B**) Sublingual DCs in CD11c-KikGR mice. The numbers indicate the frequencies of the gated population of parent cells. The data are representative of at least three independent experiments.

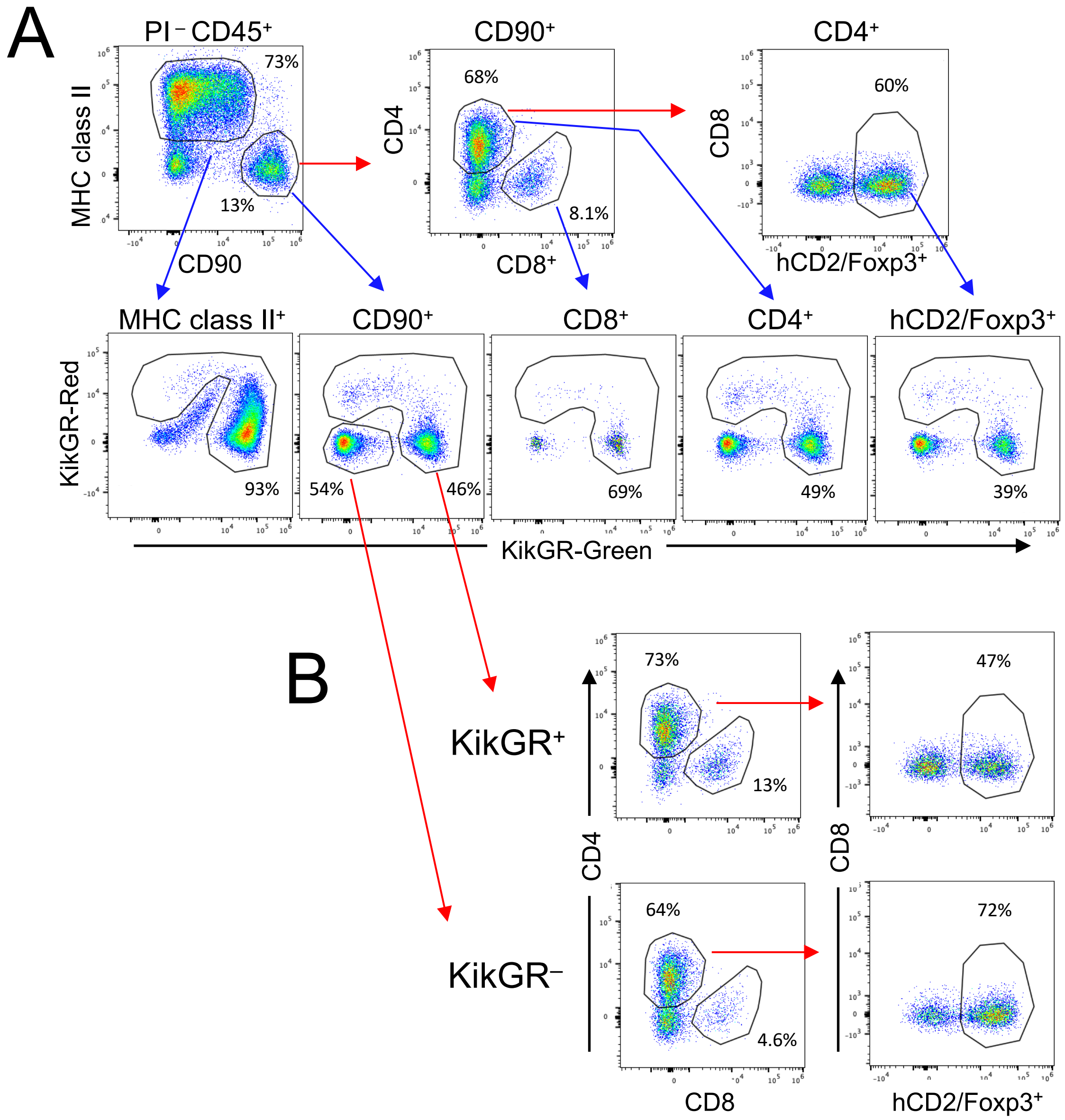

Supplemental Figure 8. Gating strategy for flow cytometric analysis and chimerism rates of sublingual T cells in hCD2/CD52-Foxp3/KikGR mouse bone marrow chimera mice. Related to Figure 7 and 8. (**A**) Gating strategy for chimerism rates of sublingual cells. Sublingual $PI^{-}CD45^{+}$ cells of hCD2/CD52-Foxp3/KikGR mouse bone marrow chimera mice were gated to MHC class II^{+} cells and $CD90^{+}$ cells. $CD90^{+}$ cells were further gated to show $CD8^{+}$ cells and $CD4^{+}$ cells, and $CD4^{+}$ cells were gated to show hCD2/$Foxp3^{+}$ cells (upper panel). The proportions of donor-derived $KikGR^{+}$ cells and host-derived $KikGR^{-}$ cells in each gated subset are shown (lower panel). (**B**) Proportions of $CD8^{+}$ cells, $CD4^{+}$ cells, and hCD2/$Foxp3^{+}$ cells in donor-derived $KikGR^{+}$ cells and host-derived $KikGR^{-}$ cells. The numbers indicate the frequencies of the gated population of parent cells. The data are representatives of at least three independent experiments.

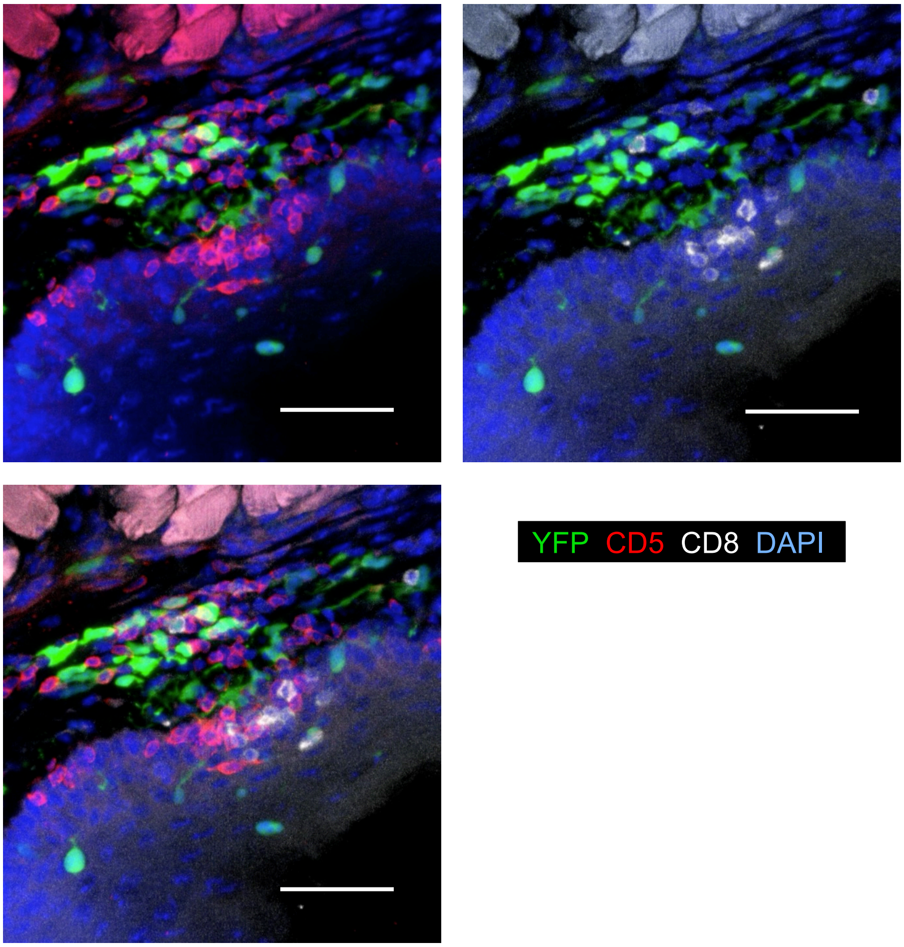

Supplemental Figure 9. Most CD8$^+$ T cells are located in epithelium in sublingual DC clusters. Related to Figure 7F, G. Images of a frozen section from sublingual tissue stained with APC conjugated anti-CD8 mAb (white), PE-conjugated anti-CD5(red), and DAPI (blue) (Supplemental Movie 9). The data are representatives of at least three independent experiments. Scale bars = 50 µm.

Supplemental Movie 9 Immunohistostaining of CD8$^+$ cells and CD5$^+$ cells in the sublingual DC cluster of CD11c-YFP mouse. Related to Supplemental Figure 9.

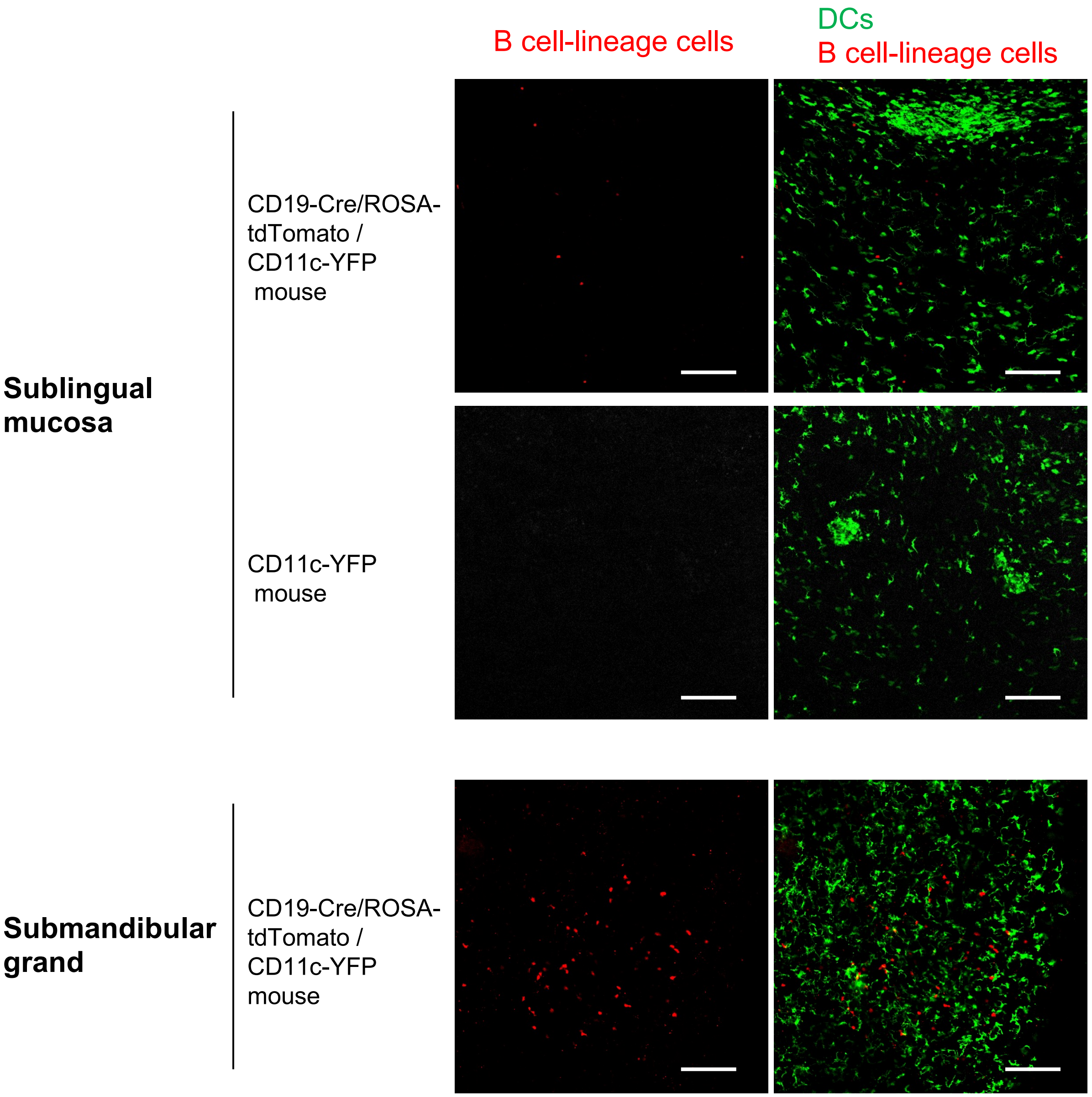

Supplemental Figure 10. Distribution of B cell-lineage cells in sublingual mucosa. Extended depth of focus images were assembled from z-images of sublingual mucosa or submandibular grand of CD19-Cre/tdTomato/CD11c-YFP mouse or CD11c-YFP mouse. The data are representatives of at least three independent experiments. Scale bars = 100 µm.

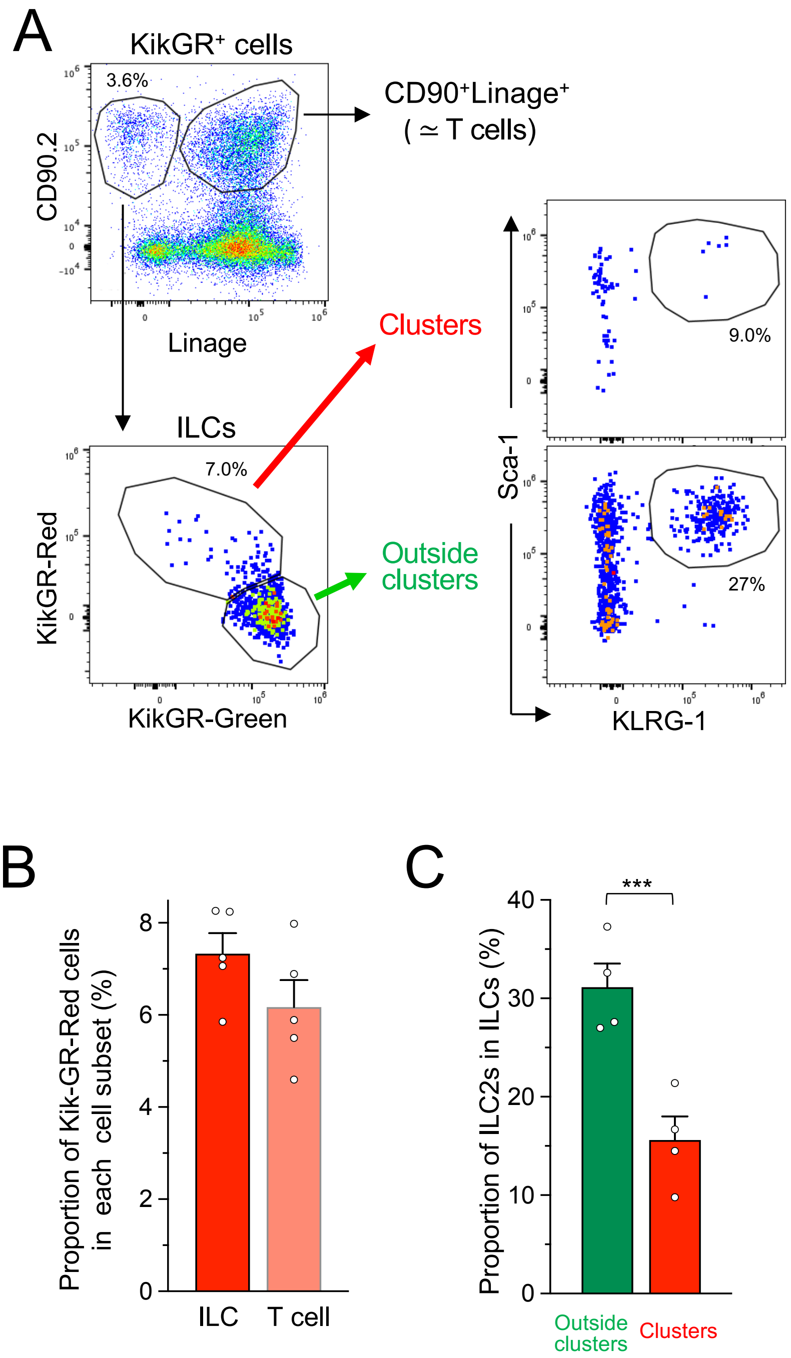

Supplemental Figure 11 Flow cytometric analysis of ILCs in SLICs in sublingual tissue. Sublingual KikGR$^+$ DC-clusters in hCD2/CD52-Foxp3/KikGR mouse bone marrow chimera mice were photo-labeled as similar to Figure 5A and single cell suspension was stained with fluorochrome antibodies. (A) Representative flow cytometry gating strategy for linage$^-$ CD90$^+$ cells (ILCs) and CD90$^+$Lineage$^+$ cells (T cells) in sublingual cells. (B) Proportion of KikGR-Red cells in ILCs, ILC2s, and T cells. (C) Proportion of ILC2s in ILCs in KikGR-Green and KikGR-Red cells. Data represent mean ± SE (n = 5). Statistical comparisons were performed using the Unpaired t-test (***p < 0.001). Lineage = CD4, CD8α, CD5, CD11b, B220, Ly6/Ly6G (Gr-1), Ter-119.

Supplemental Table 1: DC subsets and functions in human and mouse

		Langerhans cells (LC)		XCR1$^+$ DC (cDC1)	CD11b$^+$ DC
	Localization	Epithelial layer		Lamina proplia	
	Function	Intake antigen outside of tight junction		APC for CD8$^+$ T cell, cross presentation, partially Th1 induction	APC for CD4$^+$ T cell
	Molecular marker				
Mouse	EpCAM (CD326) CD1a, CD205	+		−	
Mouse	CD11b	−	+	−	+
Mouse	XCR1	+	−	+	−
Mouse	CD103	+	−	+	−
Mouse	Langerin(CD207)	+		+	−
Mouse	CD8α	−		+	−
Human	CD207, E-cadherin, CD1a, EpCAM (CD326)	+		−	
Human	CD141 (BDCA3)			+	−
Human	CD11b			−	+
Human	other positive markers			CLER9A<br>CADM1<br>XCR1<br>BTLA<br>CD26<br>DNAM-1 (CD226)	CD2<br>FCER1<br>SIRPA (CD172A)<br>ILT1<br>DCIR/CLEC4A<br>CLEC10A

Supplemental Table 2: Antibodies for Flow cytometry

moleclue	clone	fluorochrome	manufacturer
CD4	RK4-5	APC/Cy7	BioLegend
	GK5.1	PE	BioLegend
		Alexa Flour 700	BioLegend
CD5	53-7.3	PE	BioLegend
		Alexa Flour 700	BioLegend
CD8a	53-6.7	PE/Cy7	BioLegend
		APC	BioLegend
		Alexa Flour 700	BioLegend
CD11b	M1/70	PE/Cy5	BioLegend
		Alexa Flour 700	BioLegend
CD11c	N418	PE-Dazzle 594	BioLegend
CD19	6D5	PE-Dazzle 594	BioLegend
CD38	90	PE/Cy7	BioLegend
CD44	IM7	FITC	BD Phramingen
CD45	30-F11	Brilliant Violet 510	BioLegend
CD45R(B220)	RA3-6B2	APC/Cy7	BioLegend
CD62L	MEL-14	PE	BD Phramingen
CD69	H1.2F3	Brilliant Violet 421	BioLegend
CD80	16-10A1	APC	BioLegend
CD90.2	53-2.1	APC	eBioscience
		pacific blue	BioLegend
CD103	M290	APC-R700	BD Phramingen
CD127	A7R34	APC	eBioscience
CD138	281-2	Brilliant Violet 421	BioLegend
CD326	G8.8	APC/Cy7	BioLegend
Foxp3	FJK-16s	APC	eBioscience
GL7	GL7	Pacific Blue	BioLegend
Gr-1(Ly6C/Ly6G)	RB6-8C5	Alexa Flour 700	BioLegend
I-A/I-E	M5/114.15.2	Brilliant Violet 510	BioLegend
IgA	mA-6E1	PE	Invitrogen
KLRG1(MAFA)	2F1/KLRG1	Brilliant Violet 421	BioLegend
Sca1(Ly6A/Ly6E)	D7	PE/Cy5	BioLegend
TCRgd	UC7-13D5	Alexa Flour 647	BioLegend
Ter-119	TER-119	Alexa Flour 700	BioLegend
XCR1	ZET	Brilliant Violet 421	BioLegend
human CD2	RPA-2.10	Brilliant Violet 421	BioLegend
		PE/Cy5	BioLegend
